# Procedural sedation and analgesia versus general anesthesia for hysteroscopic myomectomy (PROSECCO trial): A multicenter randomized controlled trial

**DOI:** 10.1371/journal.pmed.1004323

**Published:** 2023-12-28

**Authors:** Julia F. van der Meulen, Marlies Y. Bongers, Lisa G. van der Zee, Jaklien C. Leemans, Ruben G. Duijnhoven, Robert A. de Leeuw, Lucilla E. Overdijk, Celine M. Radder, Lucet F. van der Voet, Nicol A. C. Smeets, Huib A. A. M. van Vliet, Wouter J. K. Hehenkamp, Arentje P. Manger, Arianne C. Lim, Louisette W. Peters, Nicole Horree, Justine M. Briët, Jan Willem van der Steeg, Sjors F. P. J. Coppus, Helen S. Kok

**Affiliations:** 1 Department of Obstetrics & Gynecology, Máxima Medical Centre, Veldhoven, the Netherlands; 2 Grow school for oncology and reproduction, Maastricht University Medical Centre, Maastricht, the Netherlands; 3 Faculty of Health, Medicine and Life Sciences, Maastricht University, Maastricht, the Netherlands; 4 Department of Obstetrics & Gynecology, Amsterdam University Medical Centre, Amsterdam, the Netherlands; 5 Clinical Trials Unit, Netherlands Society for Obstetrics and Gynecology, Amsterdam, the Netherlands; 6 Department of Anesthesiology, OLVG, Amsterdam, the Netherlands; 7 Department of Obstetrics & Gynecology, OLVG, Amsterdam, the Netherlands; 8 Department of Obstetrics & Gynecology, Deventer Ziekenhuis, Deventer, the Netherlands; 9 Department of Obstetrics & Gynecology, Zuyderland Medical Centre, Heerlen, the Netherlands; 10 Department of Obstetrics & Gynecology, Catharina Ziekenhuis, Eindhoven, the Netherlands; 11 Department of human structure and repair, Ghent University, Gent, Belgium; 12 Department of Obstetrics & Gynecology, Diakonessenhuis, Utrecht, the Netherlands; 13 Department of Obstetrics & Gynecology, Maastricht University Medical Centre, Maastricht, the Netherlands; 14 Department of Obstetrics & Gynecology, Flevoziekenhuis, Almere, the Netherlands; 15 Department of Obstetrics & Gynecology, Ziekenhuisgroep Twente, Almelo, the Netherlands; 16 Department of Obstetrics & Gynecology, Jeroen Bosch Ziekenhuis, ‘s-Hertogenbosch, the Netherlands; 17 Department of Obstetrics & Gynecology, University Medical Centre St Radboud, Nijmegen, the Netherlands; 18 Department of Obstetrics & Gynecology, Alrijne Ziekenhuis, Leiden, the Netherlands; University of Manchester, UNITED KINGDOM

## Abstract

**Background:**

Hysteroscopic resection is the first-choice treatment for symptomatic type 0 and 1 fibroids. Traditionally, this was performed under general anesthesia. Over the last decade, surgical procedures are increasingly being performed in an outpatient setting under procedural sedation and analgesia. However, studies evaluating safety and effectiveness of hysteroscopic myomectomy under procedural sedation are lacking. This study aims to investigate whether hysteroscopic myomectomy under procedural sedation and analgesia with propofol is noninferior to hysteroscopic myomectomy under general anesthesia.

**Methods and findings:**

This was a multicenter, randomized controlled noninferiority trial conducted in 14 university and teaching hospitals in the Netherlands between 2016 and 2021. Inclusion criteria were age ≥18 years, maximum number of 3 type 0 or 1 fibroids, maximum fibroid diameter 3.5 cm, American Society of Anesthesiologists class 1 or 2, and having sufficient knowledge of the Dutch or English language. Women with clotting disorders or with severe anemia (Hb < 5.0 mmol/L) were excluded. Women were randomized using block randomization with variable block sizes of 2, 4, and 6, between hysteroscopic myomectomy under procedural sedation and analgesia (PSA) with propofol or under general anesthesia (GA).

Primary outcome was the percentage of complete resections, assessed on transvaginal ultrasonography 6 weeks postoperatively by a sonographer blinded for the treatment arm and surgical outcome. Secondary outcomes were the surgeon’s judgment of completeness of procedure, menstrual blood loss, uterine fibroid related and general quality of life, pain, recovery, hospitalization, complications, and surgical reinterventions. Follow-up period was 1 year.

The risk difference between both treatment arms was estimated, and a Farrington–Manning test was used to determine the *p*-value for noninferiority (noninferiority margin 7.5% of incomplete resections). Data were analyzed according to the intention-to-treat principle, including a per-protocol analysis for the primary outcome.

A total of 209 women participated in the study and underwent hysteroscopic myomectomy with PSA (*n* = 106) or GA (*n* = 103). Mean age was 45.1 [SD 6.4] years in the PSA group versus 45.0 [7.7] years in the GA group. For 98/106 women in the PSA group and 89/103 women in the GA group, data were available for analysis of the primary outcome.

Hysteroscopic resection was complete in 86/98 women (87.8%) in the PSA group and 79/89 women (88.8%) in the GA group (risk difference −1.01%; 95% confidence interval (CI) −10.36 to 8.34; noninferiority, *P* = 0.09). No serious anesthesiologic complications occurred, and conversion from PSA to GA was not required. During the follow-up period, 15 serious adverse events occurred (overnight admissions). All were unrelated to the intervention studied. Main limitations were the choice of primary outcome and the fact that our study proved to be underpowered.

**Conclusions:**

Noninferiority of PSA for completeness of resection was not shown, though there were no significant differences in clinical outcomes and quality of life. In this study, hysteroscopic myomectomy for type 0 and 1 fibroids with PSA compared to GA was safe and led to shorter hospitalization. These results can be used for counseling patients by gynecologists and anesthesiologists. Based on these findings, we suggest that hysteroscopic myomectomies can be performed under PSA in an outpatient setting.

**Trial registration:**

The study was registered prospectively in the Dutch Trial Register (NTR 5357; registration date: 11 August 2015; Date of initial participant enrollment: 18 February 2016).

## Abstract 2

Julia van der Meulen and co-workers compare procedural sedation and analgesia versus general anesthesia in women undergoing hysteroscopic myomectomy for fibroids.

## Author summary

**Why was this study done?:**

➢ Hysteroscopic myomectomy is the standard treatment for type 0 and 1 fibroids and is traditionally performed with general anesthesia (GA).
➢ Procedural sedation and analgesia (PSA) is increasingly used in gynecologic surgery.
➢ The use of PSA for hysteroscopic myomectomy compared to GA in terms of efficacy and safety has not been evaluated thoroughly.

**What did the researchers do and find?:**

➢ A randomized controlled noninferiority trial was performed comparing the use of PSA with GA for hysteroscopic myomectomy. Primary outcome was completeness of resection.
➢ Noninferiority of PSA versus GA for completeness of resection could not be demonstrated.
➢ We found no significant differences in complications, recovery, quality of life, reduction in menstrual blood loss, and surgical reinterventions.
➢ The use of PSA for hysteroscopic myomectomy appeared to lead to a shorter admission time compared to GA.

**What do these findings mean?:**

➢ Hysteroscopic myomectomy can be performed under PSA in an outpatient setting.
➢ These findings should help clinicians in counseling women undergoing hysteroscopic myomectomy.

## Introduction

Hysteroscopic myomectomy is the treatment of first choice for submucous fibroids. Traditionally this procedure was performed in an operating room with general anesthesia (GA) [11]. However, in recent decades, the development of new and smaller diameter hysteroscopic instruments (without the need for cervical dilatation) has enabled the performance of many hysteroscopic procedures in an outpatient setting away from the operating theater [4,5,12]. This development leads to shorter admission time, faster recovery, and return to work, hereby reducing costs, whereas patient satisfaction and need for perioperative analgesia remain the same [20,24]. Office myomectomy techniques without anesthesia have been described for submucous fibroids with a diameter of less than 1.5 to 2 cm [11]. Nonetheless, for procedures that do require cervical dilatation, additional analgesia is necessary in an outpatient setting [11]. However, higher incompleteness rates have been described for hysteroscopic procedures performed under local anesthesia compared to GA. In addition, conversion from local anesthesia to GA might be required due to patient’s discomfort [8,33].

Procedural sedation and analgesia (PSA) is a technique of administering sedative agents with or without analgesics to create a decreased level of consciousness while maintaining cardiorespiratory functions so that patients can comfortably undergo unpleasant procedures [1]. Propofol is an intravenous anesthetic drug that can be used for moderate or deep sedation [2]. As a result of its short acting working mechanism, it leads to a rapid induction of sedation when started and a quick recovery when administration is stopped [13]. PSA with propofol is therefore used for a wide variety of procedures outside the operating room [7,10,13,14,30].

For gynecologic procedures, less literature on the use of PSA is available. A prospective cohort that was recently published showed that the performance of several therapeutic hysteroscopic procedures—including hysteroscopic myomectomy—is safe and feasible when performed under PSA with propofol: Complication rate and incompleteness rates were low, and the majority of patients was satisfied with the procedure [9]. However, the amount of myomectomies in this cohort was low, and a randomized controlled trial (RCT) comparing hysteroscopic myomectomy under PSA with the same procedure under GA is lacking. Although the advantages of an outpatient setting are present, it could be hypothesized that PSA, due to its higher level of consciousness compared to GA, could lead to more discomfort resulting in a higher number of incomplete procedures. This RCT was performed to investigate noninferiority of PSA with propofol as compared to GA for hysteroscopic myomectomy in terms of complete fibroid resection.

## Methods

### Ethics statement

The study was conducted according to the principles of the Declaration of Helsinki (World Medical Association Declaration of Helsinki Ethical Principles for Medical Research Involving Human Subjects, Version Fortaleza, Brazil, October 2013,) and ethical approval was granted by the ethics committee of the Máxima Medical Centre in Veldhoven, the Netherlands (registration number NL54779.015.15; reference number 15.106; date of approval 14 December 2015). Written informed consent was obtained from all patients before taking part.

### Trial design

The PROSECCO study was a multicenter noninferiority randomized controlled trial. The study was performed in 14 university and teaching hospitals in the Netherlands, collaborating in the Dutch Consortium for Healthcare Evaluation and Research in Obstetrics and Gynecology, between 2016 and 2021. The study was registered prospectively in the Dutch Trial Register (NTR 5357; registration date: 11 August 2015). The full study protocol was published in 2019 [32]. During the course of the study, no major changes to the study protocol were made. Minor changes to our inclusion criteria were made (maximum number of submucous fibroids 3 instead of 2; maximum size 3.5 cm instead of 3.0 cm and inclusion of English speaking patients as well). These changes were all approved by the ethics committee of the Máxima Medical Centre in Veldhoven, the Netherlands, prior to implementation. This study is reported as per the consolidated standard of reporting trials (CONSORT) statement (S1 Consort Checklist).

### Participants

Women were found eligible for inclusion if they had a minimum age of 18 years and if symptomatic International Federation of Gynecology and Obstetrics (FIGO) type 0 or 1 fibroids [25] were present, with a maximum number of 3 type 0 or 1 fibroids and a maximum diameter of 3.5 cm. They had to be American Society of Anesthesiologists (ASA) class 1 or 2 and had to have sufficient knowledge of the Dutch or English language to comprehend the study information and questionnaires. Women with known clotting disorders or severe anemia (Hb < 5.0 mmol/L) were excluded. Eligible women were identified and informed about the study by gynecologists in the participating hospitals. They were counseled by research nurses, if available, or the local investigator, and written informed consent was obtained before randomization.

### Interventions

Patients were randomized to either PSA or GA. PSA was nonanesthesiologist-administered propofol (NAAP) sedation, administered and monitored by a qualified sedation practitioner according to the guidelines from the Health Care Inspectorate (IGZ) and Dutch Institute for Healthcare Improvement (CBO) [17]. Propofol was used for procedural sedation combined with alfentanil or remifentanil intravenously for analgesia.

GA could be inhalational or total intravenously, with the use of a laryngeal mask or endotracheal tube.

Hysteroscopic myomectomy was performed by an experienced surgeon with either a resectoscope or a morcellation device.

### Outcomes

The primary outcome was the percentage of complete resections, evaluated by transvaginal ultrasonography (TVU) (contrast sonography when TVU was inconclusive) 6 weeks postoperatively. Complete resection was defined as the absence of an intracavitary remnant of the fibroid (s) resected during hysteroscopic myomectomy. TVU was performed by an experienced sonographer or gynecologist blinded for the treatment arm and the surgery outcome. If it was concluded that resection was incomplete based on TVU, the images were adjudicated by an independent review committee, blinded for the type of anesthesia and the surgeon’s judgment on completeness during the procedure. When applicable, the result of the TVU was recorded as complete after adjudication.

Secondary outcomes were as follows: completeness of resection as judged by the surgeon during the procedure; pain (Numeric Rating Scale (NRS)) score postprocedure, at discharge and 24 hours postoperatively as measured through a self-tailored questionnaire (Appendix 1 in S1 Text); recovery and return to daily activities at 24 hours, 2 weeks and 8 weeks postoperatively as measured through the Recovery Index (RI) Questionnaire [19]; duration of hospitalization; peri- and postoperative complications until 6 weeks follow-up; the need for surgical reinterventions at 12 months follow-up as measured through a self-tailored questionnaire (Appendix 1 in S1 Text); amount of menstrual blood loss at baseline, 8 weeks and 12 months follow-up as measured through Pictorial Blood Loss Assessment Chart (PBAC) score [16]; quality of life at baseline, 24 hours, 2 weeks, 8 weeks, 6 months, 12 months, as measured through the EQ-5D-5L questionnaire [15]; Uterine Fibroids Symptoms and health-related Quality of Life (UFS-QoL) at baseline and 8 weeks follow-up as measured through the UFS-QoL questionnaire [18,31].

All questionnaires were completed online. Local research nurses in the participating hospitals were responsible for collection of baseline and follow-up data from the patients’ medical records. Serious adverse events (SAEs) were reported by the local investigator to the principal investigator, who then informed the ethics committee within 15 days.

### Sample size

Prior to the study, the incidence of incomplete resections was estimated to be 2.5% in both treatment groups based on expert opinion and previous literature [21,22,26]. An upper limit of the noninferiority margin at a risk difference of 7.5% incomplete resections was considered to prove clinical noninferiority sufficiently. With an alpha of 0.025 and accounting for a loss to follow-up rate of 10%, 206 women had to be recruited to achieve 90% power.

### Randomization and blinding

Randomization was performed by using an internet-based randomization program in a 1:1 ratio with random permuted blocks of sizes 2, 4, or 6 and was stratified by the surgical technique used (morcellation or resection). Treating physicians and patients were not blinded for the allocated treatment. However, the sonographer assessing the primary outcome 6 weeks postoperatively was blinded for the treatment arm and the surgical outcome.

### Statistical methods

Data were analyzed according to the intention-to-treat (ITT) principle. Given the noninferiority design of the study, we also performed a per-protocol (PP) for the primary outcome. Imputation of missing data was not used.

The primary outcome was evaluated by estimating the risk difference between both treatment arms, with adjustment for stratification factor (resection technique). A Farrington–Manning test was used to determine the *p*-value for noninferiority. For adjustment of stratification factor, a generalized linear model was used with identity link and binomial distribution where the stratification factor was used as a covariable. Prespecified exploratory subgroup analyses were performed for parity and fibroid size (<20/≥20 mm), based on the largest fibroid seen preoperatively when >1 fibroid was present in 1 patient.

A significance level of 0.05 was used for two-sided testing. Relative risks were estimated for categorical secondary outcomes, with 95% confidence intervals (CIs), and χ^2^ tests or Fisher’s exact tests as appropriate. Continuous data were described as means with standard deviation if normally distributed, or medians with interquartile ranges; tests for significance were *t* tests or Mann–Whitney U tests, respectively. The Hodges–Lehman estimator was used to calculate the CI for the difference in medians. PBAC scores were analyzed longitudinally using a generalized estimating equations model for repeated measures and Poisson distribution. For UFS-QoL, EQ-5D-5L, RI, and NRS scores, this was not possible since data did not fit distribution assumptions. For those outcomes, the Hodges–Lehmann estimator for difference in median between groups at the different time points with *p*-value for Mann–Whitney test was estimated. A sign test for within-group change from baseline was used.

Data were analyzed using SPSS Statistics for Windows (version 22.0; IBM, Armonk, NY). For the longitudinal analysis and the noninferiority analysis SAS (version 9.4; SAS Institute, Cary, NC) was used. Details on the statistical analyses can be found in the statistical analysis plan (Statistical Analysis Plan in S1 Text).

### Patient and public involvement

Patients were actively involved in the design of the study, through the Dutch Gynecologic Patient Organization (PGN: Patiëntenvereniging Gynaecologie Nederland), in which gynecologic patients are organized. This organization endorsed the importance of the research question and was involved in the design of the protocol, the choice of outcome measures, approved the final protocol, and expected the trial to be feasible. Once the trial has been published, participants will be informed about the results in collaboration with the patient organization.

## Results

### Participants

**Fig 1:**
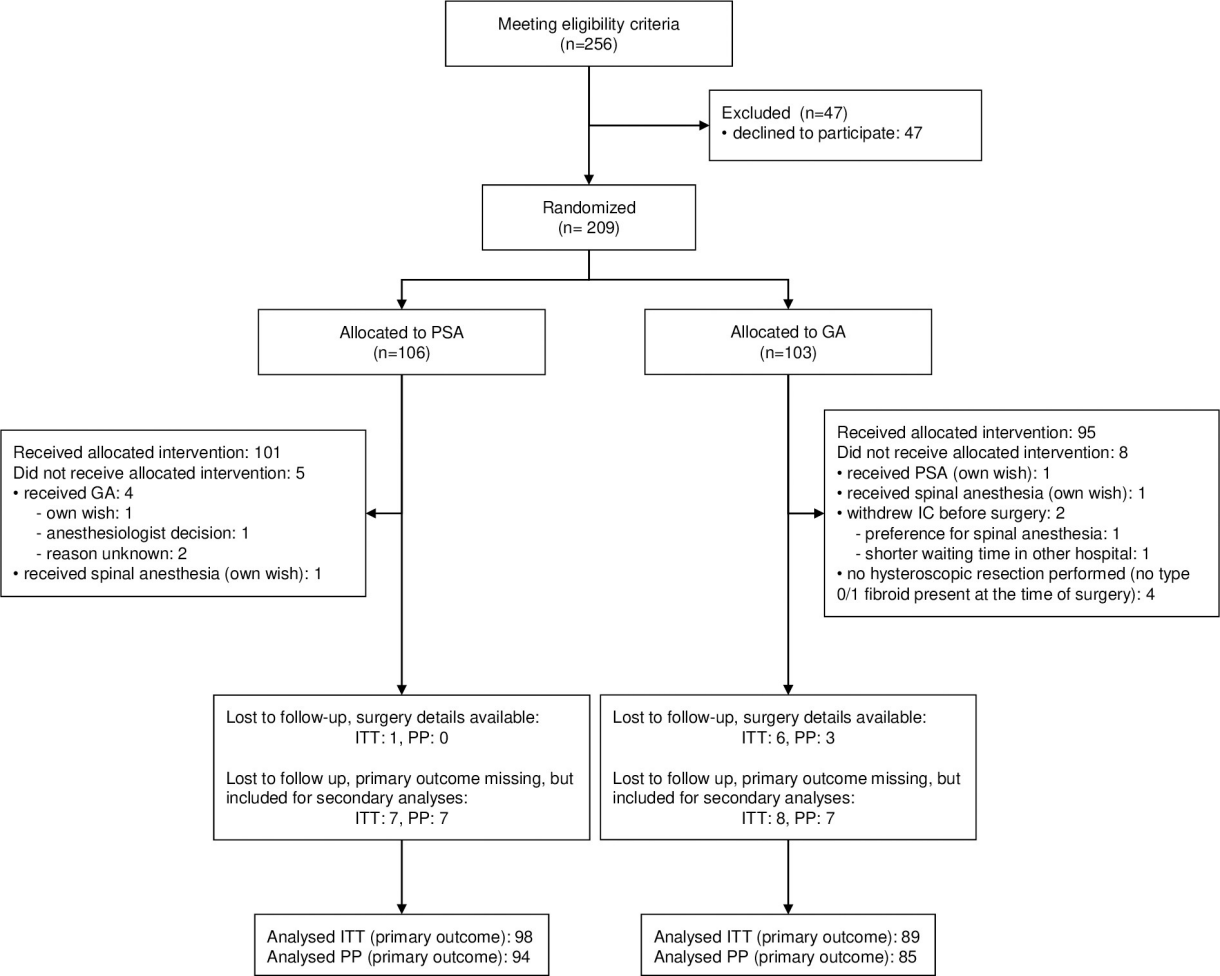
Flow diagram of PROSECCO trial. GA, general anesthesia; ITT, intention to treat; PP, per protocol; PSA, procedural sedation and analgesia. The PP population was defined as any patient who underwent myomectomy with GA or PSA as allocated using randomization. Patients who did not have a myomectomy or who did not receive the type of anesthesia allocated by randomization were removed.

Between February 2016 and December 2019, 256 women were found eligible for participation. After written informed consent was obtained, 209 women were randomly allocated to hysteroscopic myomectomy under PSA (*n* = 106) or GA (*n* = 103) (Fig 1). Due to theater waiting lists and the postponement of some hysteroscopic myomectomies due to the COVID-19 pandemic, the 1-year follow-up period for the last patient ended in April 2021.

In the PSA group, 101 of 106 women (95.3%) received the allocated intervention. PSA was administered with propofol combined with alfentanil in 72% or remifentanil in 28% of women. In the GA group, 95 of 103 women (92.2%) received GA. Reasons for deviation are presented in Fig 1. Four patients did not have hysteroscopic myomectomy due to absence of intracavitary fibroids during surgery. Therefore, we increased the number of patients recruited to correct for attrition from 206 to 209 to achieve the intended number of evaluable patients for the primary outcome (total *n* = 209).

Baseline characteristics of all women and fibroids (as examined by TVU) are presented in Table 1. In most women, only 1 fibroid was resected during hysteroscopic myomectomy. Surgical details were comparable between the 2 groups (Table A in S1 Text). When sent for histopathologic analysis, in the PSA group, a leiomyoma was confirmed in 95/105 patients (90.5%), 8 patients (7.6%) were diagnosed with a polyp, 1 patient (1.0%) with retained products of conception and 1 patient with endometrial adenocarcinoma grade 1 (1.0%). In the GA group, a leiomyoma was confirmed in 89/97 patients (91.8%). Six polyps (6.2%) and 1 polyp with atypical hyperplasia (1.0%) were diagnosed.

**Table 1:** Patient and fibroid baseline characteristics.

Characteristic	PSA N = 106	GA N = 103
Age, years (mean, sd)	45.1 (6.4)	45.0 (7.7)
BMI (median, IQR)	24.7 (22.0–28.6)	25.7 (23.4–30.5)
Parity (median, IQR)	1 (0–2)	1 (0–2)
Previous uterine surgery	18 (17.0%)	24 (23.3%)
specification:		
hysteroscopic myomectomy	8/18 (44.4%)	14/24 (58.3%)
other	10/18 (55.6%)	10/24 (41.7%)
Use of hormonal medication at the time of surgery	52 (49.1%)	45 (43.7%)
Reason for hysteroscopic myomectomy*		
abnormal uterine bleeding	101 (96.2%)	96 (93.2%)
abdominal complaints	6 (5.7%)	9 (8.7%)
subfertility	4 (3.8%)	5 (4.9%)
other	1 (1.0%)	2 (1.9%)
No. submucous myomas intended to be resected	119	113
one	93 (87.7%)	94 (91.3%)
two	13 (12.3%)	8 (7.8%)
three	0 (0.0%)	1 (1.0%)
Myoma type		
type 0	46 (39.3%)	56 (52.8%)
type 1	71 (60.7%)	50 (47.2%)
Maximum size (diameter [mm]; mean, sd)	20.9 (6.4)	19.9 (6.8)
Presence of additional fibroids that can’t be resected hysteroscopically		
yes	36 (34.6%)	30 (29.1%)

Percentages are column percentages based on the number of observations available. PSA: Procedural sedation and analgesia, GA: General anesthesia, BMI: Body Mass Index, IQR: Interquartile Range, sd: standard deviation,. *multiple reasons can apply.

### Loss of follow-up and missing data

For 8 women (7.5%) in the PSA group, the primary outcome (TVU at 6 weeks postsurgery) was missing. In the GA group, the TVU result was unavailable for 14 women (13.6%). Among these women, 7/8 in the PSA group and 8/14 in the GA group did complete the digital questionnaires and could still be included in analyses for secondary outcomes (Fig 1).

### Primary outcome

Hysteroscopic resection was complete (as based on TVU) in 86/98 (87.8%) women under PSA and in 79/89 (88.8%) under GA (risk difference −1.01%; 95% CI −10.36 to 8.34). Although the difference in completeness between both groups was very small, significant noninferiority could not be demonstrated (*p*-value 0.09). When incomplete, the diameter of the intracavitary remnant did not differ significantly between the PSA and GA group (*p*-value 0.15). The PP analysis result was comparable to the ITT analysis (Table 2).

**Table 2:** Completeness of resection based on TVU 6 weeks after hysteroscopic myomectomy.

	ITT				PP			
	PSA *N* = 98	GA *N* = 89	Risk diff. (95% CI)	*P* value	PSA *N* = 94	GA *N* = 85	Risk diff. (95% CI)	*P* value
Resection complete*	86 (87.8%)	79 (88.8%)	−1.01% (−10.36–8.34)	0.09	83 (88.3%)	75 (88.2%)	0.06% (−9.53–9.65)	0.06
adjusted for stratification factors			−1.83% (−11.2–7.5)	-			0.82% (−8.63–10.28)	-
When incomplete; max. size of intracavitary remnant (mean SD)†	23.9 (13.7)	16.2 (6.9)	7.7 (−3.2–18.5)	0.15	24.6 (14.4)	16.2 (6.9)	8.4 (−3.1–19.9)	0.14
<5 mm	0	0			0	0		
≥5–10 mm	1	1			1	1		
≥10–15 mm	1	3			1	3		
≥15–20 mm	3	3			2	3		
≥20–30 mm	2	2			2	2		
≥30–40 mm	0	0			0	0		
≥40 mm	2	0			2	0		
unknown	3	1			3	1		

CI, confidence interval; GA, general anesthesia; PP, per protocol; PSA, procedural sedation and analgesia; SD, standard deviation; TVU, transvaginal ultrasound.

Percentages are column percentages based on the number of observations available (i.e., excluding missing observations).

*Risk difference with *p*-value of Farrington–Manning test for noninferiority against a margin of −7.5%.

^†^Mean difference and *p*-value for independent sample *t* test.

In the subgroup analyses performed for parity (nulliparous versus primi- and multiparous) and fibroid size (<20 mm versus ≥20 mm), significant subgroup effects were demonstrated for parity (*p*-value for interaction 0.029; Table B in S1 Text).

### Secondary outcomes

There was no significant difference in surgeon’s judgment on completeness of resection in the PSA group compared to the GA group (79/106 (74.5%) versus 82/99 (82.8%), RR 0.90; 95% CI 0.78 to 1.04; *p* = 0.15). Easiness of the procedure did not differ significantly (Table C in S1 Text). When incomplete, a second hysteroscopic myomectomy session was performed in 12/27 women (44.4%) in the PSA group, for which complete fibroid removal could be achieved in 9/12 women (75%). In the GA group, a second session was performed in 7/17 women (41.2%) with completeness achieved in 5/7 women (83.3%). A third planned session for fibroid removal was not required in either of the groups. Reasons for incompleteness are shown in Table C in S1 Text.

A significant difference in median NRS score was observed between the PSA group and GA group in favor of the GA group immediately after surgery in the recovery room (2.0 (0.0 to 4.0) versus 0.0 (0.0 to 2.0); median difference 0.0; 95% CI 0.0 to 1.0; *p*-value 0.027). However, this difference was no longer significant at discharge and at 24 hours follow-up (Table D in S1 Text).

Recovery, as measured through the RI Questionnaire, did not differ significantly between the PSA and GA group at any follow-up moment (Table E in S1 Text).

Median duration of hospital admission was significantly shorter in the PSA group compared to the GA group: 240.5 (185.0 to 335.0) minutes versus 386 (320.0 to 447.0) minutes; median difference −140.0; 95% CI −169.0 to −109.0; *p* < 0.0001.

Surgical perioperative complications occurred in 8/106 (7.5%) women under PSA and 5/101 (5.0%) women under GA (RR 1.53; 95% CI 0.52 to 4.51; *p*-value 0.44) (Table F in S1 Text). Anesthetic complications needing intervention included desaturation, airway obstruction, and blood pressure drops and occurred in 5/106 (4.7%) women in the PSA group and 4/101 (4.0%) of women in the GA group (RR 1.19; 95% CI 0.33 to 4.31, *p*-value 1.00) (Table 3). Conversion from PSA to GA during the procedure was never required. There was no significant difference in postoperative complications (Table G in S1 Text).

**Table 3:** Anesthetic complications.

Anesthetic complications	PSA *N* = 106	GA *N* = 101	Rel. risk (95% CI)	*P* value
Anesthetic complication needing intervention	5 (4.7%)	4 (4.0%)	1.19 (0.33–4.31)	1.00
if complication, specification:				
desaturation	3/5 (60.0%)	1/4 (25.0%)		
airway obstruction	2/5 (40.0%)	0/4 (0.0%)		
hypotension	0/5 (0.0%)	3/4 (75.0%)		
Perioperative nausea/vomiting/aspiration	0/5 (0.0%)	0/4 (0.0%)		
Treatment of complication*:				
supplemental oxygen	2/5 (40%)	0/4 (0.0%)		
airway maneuver	4/5 (80%)	0/4 (0.0%)		
naso-/oropharyngeal airway	1/5 (20%)	0/4 (0.0%)		
Intubation	0/5 (0.0%)	1/4 (25%)		
medication given*:	0/5 (0.0%)	3/4 (75%)		
fenylefrine	0	3		
ephedrine	0	2		

CI, confidence interval; GA, general anesthesia; PSA, procedural sedation and analgesia.

Percentages are column percentages based on the number of observations available (i.e., excluding missing observations). Relative risk with 95% CI and chi-squared test.

*Multiple answers can apply.

There was no significant difference in surgical reinterventions in the PSA group compared to the GA group at 12 months follow-up (8/77 (10.4%) versus 9/68 (13.2%); RR 0.79; 95% CI 0.32 to 1.92; *p*-value 0.60). Data on surgical reinterventions are presented in Table 4, showing that a hysterectomy was the most common reintervention.

**Table 4:** Surgical reinterventions.

Surgical reintervention	PSA N = 77	GA N = 68	Rel. risk (95% CI)	P-value
Reintervention	8 (10.4%)	9 (13.2%)	0.79 (0.32–1.92)	0.60
Hysteroscopic myomectomy	1/8 (12.5%)	1/9 (11.1%)		
Endometrial ablation	1/8 (12.5%)	2/9 (22.2%)		
Hysterectomy	5/8 (62.5%)	5/9 (55.6%)		
Sonata (RF ablation)	1/8 (12.5%)	0/9 (0.0%		
Laparoscopic myomectomy	0/8 (0.0%)	1/9 (11.1%)		

PSA: Procedural sedation and analgesia, GA: general anesthesia, RF: radiofrequency. Percentages are column percentages based on the number of observations available. Relative risk with 95% confidence interval and Chi-squared test.

PBAC score, EQ-5D-5L score, and UFS-QoL score significantly improved over time compared to baseline within the groups with no significant differences between the PSA and GA group (Table H, I, and J in S1 Text).

During the study, 15 SAEs occurred. An overview is provided in Table 5. They were all assessed as unrelated to the intervention studied.

**Table 5:** Serious adverse events.

SAE		PSA	GA
		*N* = 106	*N* = 101
Hysterectomy with overnight admission		5 (4.7%)	5 (5.0%)
	endometriumcarcinoma	1	
	polyp with atypical hyperplasia		1
	leiomyoma with pathological features suspicious of leiomyosarcoma		1
	heavy menstrual bleeding (no fibroids)	2	1
	fibroid related complaints	2	2
New hysteroscopic myomectomy after perforation in first session			1 (1.0%)
Heavy bleeding with overnight admission		3 (2.8%)	
	miscarriage related; bloodtransfusion	1	
	abortion related; curettage	1	
	heavy menstrual bleeding, no fibroid present; bloodtransfusion	1	
Cerebrovascular accident with overnight admission			1 (1.0%)
Total		8 (7.5%)	7 (6.9%)

GA, general anesthesia; PSA, procedural sedation and analgesia; SAE, serious adverse event.

Values are presented as number of women with percentage.

## Discussion

### Main findings

This multicenter RCT comparing PSA with GA for hysteroscopic myomectomy found similar percentages for completeness of fibroid resection in both groups (87.8% and 88.8%). However, significant noninferiority could not be demonstrated. Among secondary endpoint findings, hysteroscopic myomectomy appeared to lead to a considerable shorter hospital admission duration when performed under PSA. Immediately after surgery, women in the GA group reported a lower NRS score than women in the PSA group. However, at discharge and at 24 hours follow-up, NRS score was not significantly different anymore. No significant differences were found for other secondary outcomes. All complications that occurred were minor. Conversion from PSA to GA was not required.

This is the first RCT comparing PSA with GA for hysteroscopic myomectomy. To our knowledge, there are no other trials that report on outcomes of PSA for hysteroscopic myomectomy alone. The largest prospective cohort study on PSA with propofol for therapeutic hysteroscopic procedures was performed by Cornelissen and colleagues, who described the results of 455 hysteroscopic procedures [9]. In 3.3% of the patients, the procedure was incomplete, and in 6.2% of patients, a reintervention was needed. Because in this study only 6% of the 455 procedures were myomectomies (*n* = 27), it is difficult to compare their results with those of our study. Their overall anesthetic complication rate (3.5%), however, was comparable to our results (PSA group 4.7%, GA group 4.0%). Only a few other studies report on use of PSA with propofol for gynecologic surgery, including hysteroscopic polypectomies[3,6], thermal balloon ablation[23], vaginal prolapse surgery[27,29], and laparoscopic procedures (mainly salpingo-oophorectomies and laser ablation of endometriosis) [28]. Since these procedures are different from our study, it is difficult to compare completeness rates. Nevertheless, these studies report no major complications and show that the use of PSA with propofol for these procedures is safe and feasible.

### Strengths and weaknesses of the study

An important strength of this study is that it is the first RCT on PSA versus GA for hysteroscopic myomectomy. Secondly, it was a multicenter study, in which 14 hospitals in the Netherlands participated, resulting in generalizable results. Thirdly, sonographers evaluating the primary outcome were blinded for the treatment arm and surgeon’s evaluation, resulting in objective assessment of the primary outcome. Lastly, not only completeness of resection and safety were evaluated, but also other outcomes were assessed, such as (uterine fibroid related) quality of life, menstrual blood loss, and recovery with a 1-year follow-up period. Therefore, this study provides important information on the use of PSA for hysteroscopic myomectomy.

The most important limitations were the choice of primary outcome, and the fact that our study proved to be underpowered. At design of the trial, it was hypothesized that PSA could lead to lower patient comfort and hereby inability to complete the procedure. Therefore, an outcome parameter as objective as possible for complete fibroid resection (TVU by a blinded sonographer) was chosen. However, it turned out that TVU after 6 weeks was not always accurate to evaluate completeness and success of the procedure. For example, when multiple fibroids were present, it was difficult for a blinded sonographer to assess if there was a remnant of a resected fibroid or if these fibroids had not been resected and were still expected to be present. Also, the presence of a small remnant lesion without any clinical consequences does not necessarily mean that the procedure was unsuccessful from a patient perspective. In retrospect, one could argue that reduction in menstrual bleeding or improvement in quality of life would have been a better primary outcome.

Although the percentage of complete resections was similar in both groups, noninferiority was not demonstrated, as the study did not achieve the planned power of 90%. Based on expert opinion and literature, we estimated the percentage of incomplete resections at 2.5%. However, the use of different outcomes (such as surgical reintervention, reduction in bleeding, recurrence of fibroids or symptoms) for the definition of successful hysteroscopic myomectomy made it difficult to estimate this percentage. The actual percentage of incomplete resections in our study, however, was 11.8%. Therefore, the required sample size should have been larger to possibly demonstrate noninferiority. It is, however, reasonable to argue that the estimated risk difference of −1.01% between PSA and GA for completeness of resection is irrelevant from a clinical perspective. Moreover, the 95% CI of −10.36 to 8.34 shows that the outcome of PSA and GA is very comparable, in which hysteroscopic myomectomy under PSA could be up to 8% more effective and 10% less effective, a margin which—in many other studies—is often found acceptable.

Despite the study’s power limitation, the Data and Safety Monitoring Board (DSMB) advised to continue the study as planned, since it would provide important data on many other outcomes and changing the primary outcome during the study period would have reduced the methodological quality.

### Unanswered questions and future research

Since hysteroscopic myomectomy under PSA reduces hospital admission duration in comparison to GA, it can be assumed to result in a reduction of overall costs. Alongside this study, we conducted a cost effectiveness study, of which results will be published at a later stage.

Although quality of life did improve (both general and uterine fibroid related) and was not significantly different in the PSA and GA group, we did not directly address patient satisfaction in our study. Therefore, it would be advisable for future studies to address patient satisfaction as well.

## Conclusions

Noninferiority of PSA to GA for completeness of resection could not be demonstrated. However, there were no differences in clinical outcomes and quality of life. In this study, hysteroscopic myomectomy for type 0 and 1 fibroids with PSA compared to GA was safe and led to shorter hospitalization. Gynecologists and anesthesiologist can use these results to counsel patients who will undergo hysteroscopic myomectomy. Based on these results, we suggest that hysteroscopic myomectomies can be performed under PSA in an outpatient setting.

## Supporting information

S1 Consort Checklist CONSORT 2010 checklist of information to include when reporting a randomised trial. (DOC)

S1 Protocol Prosecco trial. (PDF)

S1 Text Supporting information PROSECCO trial. Appendix 1. Questionnaires. Vragenlijst bijwerkingen 24 uur na hysteroscopische myoomresectie (Dutch version). Questionnaire on side effects 24 hours after hysteroscopic myomectomy (English version). Vragenlijst evaluatie recidief na hysteroscopische myoomresectie (Dutch version). Questionnaire for evaluation of recurrence after hysteroscopic myomectomy (English version). Appendix 2. Supplementary tables. Table A. Surgical and anesthesia details. Table B. Subgroup analyses for primary outcome. Table C. Surgeon’s judgment on completeness and ease of procedure. Table D. NRS score. Table E. Recovery Index Questionnaire. Table F. Surgical complications. Table G. Postoperative complications. Table H. PBAC score. Table I. EQ-5D-5L. Table J. UFS-QoL. Appendix 3. Statistical Analysis Plan.
(PDF)

## Abbreviations

ASA: American Society of Anesthesiologists
CI: confidence interval
DSMB: Data and Safety Monitoring Board
FIGO: International Federation of Gynecology and Obstetrics
GA: general anesthesia
ITT: intention to treat
NAAP: nonanesthesiologist-administered propofol
NRS: Numeric Rating Scale
PBAC: Pictorial Blood Loss Assessment Chart
PP: per-protocol
PSA: procedural sedation and analgesia
RCT: randomized controlled trial
RI: Recovery Index
SAE: serious adverse event
TVU: transvaginal ultrasonography
UFS-QoL: Uterine Fibroids Symptoms and health-related Quality of Life
