# Supplementary material for: Procedural sedation and analgesia versus general anesthesia for hysteroscopic myomectomy (PROSECCO trial): A multicenter randomized controlled trial
Source: PLoS Med. 2023 Dec 28;20(12):e1004323. doi: 10.1371/journal.pmed.1004323 (PMC10754450; doi:10.1371/journal.pmed.1004323)
Supplement: S1 Protocol — (PDF) [file pmed.1004323.s002.pdf]

PROSECCO TRIAL

RESEARCH PROTOCOL

Procedural sedation for hysteroscopic myomectomy: cost effectiveness

PROTOCOL TITLE

Procedural sedation for hysteroscopic myomectomy – cost effectiveness (Prosecco)

**Protocol ID**	**NL54779.015.15**
**Short title**	**Prosecco trial**
**Version**	**2**
**Date**	**17-11-2015**
**Coordinating investigator/project leader**	Dr. H.S. Kok<br>Alrijne Ziekenhuis Leiden<br>Houtlaan 55<br>2334 CK LEIDEN<br>T: 071-5178178<br>E: hskok@diaconessenhuis.nl
**Principal investigator(s) (in Dutch: hoofdonderzoeker/ uitvoerder)**	Prof. Dr. M.Y. Bongers<br>Máxima Medisch Centrum<br>Department of obstetrics and gynaecology<br>De Run 4600<br>5504 DB VELDHOVEN<br>T: 040-8888000<br>E: my.bongers@gmail.com
**Fellow investigator**	J.F. van der Meulen<br>Máxima Medisch Centrum<br>Department of obstetrics and gynaecology<br>De Run 4600<br>5504 DB VELDHOVEN<br>T: 040-8888000<br>E: julia.vander.meulen@mmc.nl
Participating centers	Academisch Medisch Centrum Amsterdam<br>Prof. Dr. J.P.W.R. Roovers<br><br>Onze Lieve Vrouwe Gasthuis Amsterdam<br>Dr. E.A. Bakkum<br><br>Vrije Universiteit Medisch Centrum Amsterdam<br>Dr. W.J.K. Hehenkamp<br><br>Sint Lukas Andreas Ziekenhuis Amsterdam<br>Dr. C.M. Radder<br><br>Flevo Ziekenhuis Almere<br>Dr. W.M. van Baal

	Màxima Medisch Centrum Veldhoven<br>Prof. Dr. M.Y. Bongers<br><br>Catharina Ziekenhuis Eindhoven<br>Dr. H.A.A.M. van Vliet<br><br>Maastricht Universitair Medisch Centrum Maastricht<br>Dr. W.A. Spaans<br><br>Radboud Universitair Medisch Centrum Nijmegen<br>Dr. S.F.P.J. Coppus<br><br>Deventer Ziekenhuis<br>L.F van der Voet<br><br>Zuyderland Medisch Centrum<br>Dr. N.A.C. Smeets
**Sponsor**	Máxima Medisch Centrum<br>Postbus 7777<br>5500 MB VELDHOVEN<br>T: 040-8888000
**Subsidising party**	ZonMW<br>Postbus 93 245<br>2509 AE Den Haag<br>T: 070-3495111<br>E: info@zonmw.nl
**Independent expert (s)**	Dr. R. de Jongh<br>urologist<br>De Run 4600<br>5504 DB VELDHOVEN<br>T: 040-8888000<br>E: Rik.de.Jongh@mmc.nl

PROTOCOL SIGNATURE SHEET

**Name**	**Signature**	**Date**
**Principal Investigator:**<br>**Prof. Dr. M.Y. Bongers**		23-11-15

TABLE OF CONTENTS

1. INTRODUCTION AND RATIONALE 7
2. OBJECTIVES 11
3. STUDY DESIGN 12
4. STUDY POPULATION 13
   4.1 Population (base) 13
   4.2 Inclusion criteria 13
   4.3 Exclusion criteria 13
   4.4 Sample size calculation 13
5. TREATMENT OF SUBJECTS 14
   5.1 Investigational product/treatment 14
6. METHODS 15
   6.1 Study parameters/endpoints 15
      6.1.1 Main study parameter/endpoint 15
      6.1.2 Secondary study parameters/endpoints 15
   6.2 Randomisation, blinding and treatment allocation 15
   6.3 Study procedures 15
   6.4 Withdrawal of individual subjects 17
7. SAFETY REPORTING 18
   7.1 Section 10 WMO event 18
   7.2 AEs, SAEs 18
      7.2.1 Adverse events (AEs) 18
      7.2.2 Serious adverse events (SAEs) 18
   7.3 Follow-up of adverse events 19
   7.4 Data Safety Monitoring Board (DSMB) 19
8. STATISTICAL ANALYSIS 20
   8.1 Data analysis and presentation/synthesis 20
   8.2 Cost effectiveness analysis (CEA) 20
   8.3 Budget Impact Analysis (BIA) 21
   8.4 Interim analysis 22
9. ETHICAL CONSIDERATIONS 23
   9.1 Regulation statement 23
   9.2 Recruitment and consent 23
   9.3 Benefits and risks assessment, group relatedness 23
   9.4 Compensation for injury 23
10. ADMINISTRATIVE ASPECTS, MONITORING AND PUBLICATION 24
   10.1 Handling and storage of data and documents 24
   10.2 Amendments 24
   10.3 Annual progress report 24
   10.4 End of study report 24
   10.5 Public disclosure and publication policy 25
11. REFERENCES 26

LIST OF ABBREVIATIONS AND RELEVANT DEFINITIONS

AE	Adverse Event
AR	Adverse Reaction
CA	Competent Authority
CBO	Dutch Institute for Healthcare Improvement
CCMO	Central Committee on Research Involving Human Subjects; in Dutch: Centrale Commissie Mensgebonden Onderzoek
CEA	Cost effectiveness analysis
CV	Curriculum Vitae
DSMB	Data Safety Monitoring Board
EU	European Union
GCP	Good Clinical Practice
IC	Informed Consent
IGZ	Health Care Inspectorate
iMCQ	Medical Consumption Questionnaire
iPCQ	Productivity Cost Questionnaire
METC	Medical research ethics committee (MREC); in Dutch: medisch ethische toetsing commissie (METC)
NAAP	Non-Anesthesiologist Administered Propofol
PBAC	Pictorial Blood Assessment Chart
PSA	Procedural sedation and analgesia
RCT	Randomized Controlled Trial
(S)AE	(Serious) Adverse Event
SF-HLQ	Short Form-Health and Labour Questionnaire
Sponsor	The sponsor is the party that commissions the organisation or performance of the research, for example a pharmaceutical company, academic hospital, scientific organisation or investigator. A party that provides funding for a study but does not commission it is not regarded as the sponsor, but referred to as a subsidising party.
SUSAR	Suspected Unexpected Serious Adverse Reaction
TVU	Transvaginal Ultrasonography
UFS-QoL	Uterine Fibroid Symptoms – Quality of Life questionnaire
WMO	Medical Research Involving Human Subjects Act (in Dutch: Wet Medisch-wetenschappelijk Onderzoek met Mensen

SUMMARY

Rationale: Hysteroscopic myomectomies are performed in the majority of Dutch hospitals. The number of procedures for submucosal type 0 or I myomas between 1-3 cm performed in the operating room is estimated to be 3000 per year. Hysteroscopic myomectomy is currently performed in daycare under general anesthesia. A considerable cost reduction is expected when procedural sedation (PSA) is applied. The major contributing factors to the cost reduction are the shift from surgery in an operating theatre to an office-based setting, shorter hospital stay and outpatient care versus day care. We expect higher patient satisfaction, as hospital stay is shorter and side effects such as nausea are reduced. However, both safety and effectiveness – including the necessity for re-intervention due to incomplete resection – have not yet been fully evaluated.

Objective: Is hysteroscopic resection of submucous myomas under procedural sedation with propofol (PSA) in an outpatient setting a cost-effective alternative for hysteroscopic myomectomy performed under general anesthesia in the operation room?

Study design: non-inferiority, multicenter RCT, with a cost effectiveness analysis (CEA) alongside it.

Study population: patients with submucosal type 0 or I myomas (FIGO (PALM COEIN)) with a maximum diameter of 3 cm. We use a non-inferiority design: based on estimated risk of 2,5% incomplete resections in both groups, with an non-inferiority upper limit of 10%, 205 women are needed.

Intervention: hysteroscopic resection of submucous myomas under procedural sedation with propofol, compared to general anesthesia

Main study parameters/endpoints: 1) complete resection 2) costs, pain, menstrual blood loss (PBAC score), , quality of life, return to daily activities/work, hospitalization, (post)operative complications, re-interventions.

Nature and extent of the burden and risks associated with participation, benefit and group relatedness: Patients participating in this study are requested to complete a questionnaire at baseline, after 24 hours, 2 weeks, 8 weeks, 6 months and 12 months after the procedure. At 6 weeks after the procedure patients are asked to visit the outpatient department for a transvaginal ultrasound to assess the completeness of resection. This visit is part of the current usual care.

1. INTRODUCTION AND RATIONALE

Myomas are a frequent finding in patients with abnormal uterine bleeding. Hysteroscopic resection of submucosal myomas is an effective therapeutic intervention in case of heavy menstrual bleeding which is performed in an estimated 3000 women annually in the Netherlands. This number is extrapolated from data obtained from annual reports of 15 Dutch hospitals (teaching and non-teaching hospitals). A success rate (reduction of symptoms) of 70-99% has been reported [1-6]; the wide range reflects inclusion of all types of submucous myomas, also intramural myomas (FIGO PALM-COEIN type 2 submucous myomas) in the studies. The Dutch guideline on Heavy Menstrual Bleeding recommends hysteroscopic myomectomy as a first choice treatment in case of submucosal myomas.
In the last decades, there has been a trend in hysteroscopic surgery to move from a traditional operating theatre with general anesthesia to an outpatient setting. This trend has specifically been observed for hysteroscopic surgery of smaller diameter polyps (< 2 cm) and low grade adhesions [7-9]. Even for smaller type 0 and 1 submucosal myomas it has been reported that these can successfully be removed in an outpatient setting without anesthesia[10]. Larger myomas generally require the use of a larger diameter instrument and hence the need of cervical dilatation and general anesthesia.
Procedural sedation is used for a wide variety of interventional procedures in multiple settings outside the operation room. There is literature examining safety and efficacy of procedural sedation during gastrointestinal endoscopic procedure, emergency department procedures and pediatric sedation. [11-15].
In gynecology, the use of procedural sedation has also become more popular since technical and instrumental improvements have significantly increased the feasibility and acceptability of hysteroscopy in outpatient settings [16-17]. For resection of myomas, procedural sedation is currently not commonly used. Potential advantages of hysteroscopic resection of myomas with procedural sedation are avoidance of general anesthesia and its associated risks and side effects, a shorter recovery time, a shorter hospital stay and return to mobility and full fitness, faster return to normal activities and work, lower costs related to decreased use of dedicated personnel and operating room, and reduced waiting lists for major surgery by averting the need for the operating room for minor procedures [18].

HEALTH CARE EFFICENCY PROBLEM: Hysteroscopic myomectomies are performed in the majority of Dutch hospitals. The number of procedures for submucosal type 0 or I myomas between 1-3 cm performed in the operating room is estimated to be 3000 per year. This number is extrapolated from data obtained from annual reports of 15 Dutch hospitals (teaching and non-teaching hospitals). Hysteroscopic myomectomy is currently performed in

daycare under general anesthesia. A considerable cost reduction is expected when procedural sedation is applied. The shift from surgery in an operating theatre to an office-based setting and shorter hospital stay -day care versus outpatient care- are the major contributing factors to the cost reduction. We expect higher patient satisfaction, as both hospital stay and time-to-work are shorter and side effects such as nausea are reduced. However, both safety and effectiveness – including the necessity for re-intervention due to incomplete resection- have not yet been fully evaluated. In summary, we expect comparable effectiveness of the procedure in a safe and patient friendly environment whilst achieving a significant cost reduction.

USUAL/STANDARD CARE: The Dutch Guideline 'Heavy menstrual bleeding' (NVOG: Dutch Society of Obstetrics and Gynecology) recommends hysteroscopic myomectomy as treatment of first choice in case of symptomatic submucous myomas [19]. In the current situation, patients are admitted into daycare and they are operated under general anesthesia. This requires an operating room with a full anesthetic team. Postoperatively, patients are transferred to the recovery unit from which they go back to the ward. Several hours later discharge follows. Procedural sedation has never been evaluated for hysteroscopic myomectomy regarding (cost)effectiveness. The guideline committee suggests that local anesthesia or 'conscious sedation' can be taken into consideration for hysteroscopic myomectomy based on indirect evidence from other areas in healthcare [19].

Relevance

THE INTERVENTION TO BE INVESTIGATED: Hysteroscopic myomectomy for symptomatic type 0 or I submucosal myomas in an outpatient setting using procedural sedation with propofol compared to hysteroscopic myomectomy in an inpatient clinical setting using general anesthesia. According to the guidelines from the Health Care Inspectorate (IGZ) and Dutch Institute for Healthcare Improvement (CBO) procedural sedation is administered by a qualified sedation practitioner with standard monitoring.

EXISTING EVIDENCE OF EFFECTIVENESS: Due to proved safety, higher patient satisfaction, shorter procedure time and faster recovery the use of propofol in sedation practice has increased [11-12; 20]. Analogue to the successful shift of diagnostic procedures to an office-based setting [21-22], hysteroscopic myomectomies can be moved away from the operating theatre when adequate sedation and analgesia is secured. In a systematic review we found two studies evaluating feasibility and patient satisfaction with gynaecological surgery under procedural sedation [23-24]. From these studies, sedation with propofol/alfentanil seems to be feasible for endometrial balloon ablation and prolaps surgery.

In a second systematic review we evaluated the outcome of hysteroscopic resection of type 0 and type 1 myomas. The results of hysteroscopic removal of type 0/1 are very good with complete removal up to 100% and successful reduction of menstrual bleeding. The risk of complications is low (3,2% in a prospective bipolar resection trial of 449 patients) [25].
In summary, the systematic review evidence suggests that 1. procedural sedation anesthesia is feasible and well-tolerated in gynaecological surgery; and 2. hysteroscopic myomectomy is a safe and technically successful procedure. However, there are no randomised efficacy data.

INNOVATIVE CHARACTER: The proposed RCT is the first trial comparing the use of PSA in hysteroscopic myomectomy compared with the current standard hysteroscopic myomectomy in an inpatient setting under general anesthesia.

RELEVANCE FOR PRACTICE: The Dutch Guideline 'Heavy menstrual bleeding' recommends hysteroscopic myomectomy as treatment of first choice in case of symptomatic submucous myomas. In the current situation, patients are admitted into daycare and they are operated under general anesthesia. Performing hysteroscopic myomectomy under propofol sedation will lead to numerous advantages. For the patients direct advantages are a shorter hospital stay, less risk related to general anesthesia, treatment in a patient friendly setting and adequate recovery to pre-procedural functional and physiological level. For the hospital organization it leads to less pressure on surgery waiting lists, more flexible scheduling and a cost-reduction. From a societal perspective a further cost reduction is expected because of faster return to normal activities and shorter absence of work.

ANTICIPATED COST-EFFECTIVENESS: With an incidence of 3000 myomas eligible to resect in an office setting annually, and an estimated cost saving of €595 per resection, an estimated cost reduction of almost 1,8 million euros can be expected from a healthcare perspective annually. When taking cost savings due to reduced productivity losses into account, the savings are around 2,8 million euros annually from a societal perspective. Not unimportant to add is that we expect this cost-reduction at a concurrent improvement in patient satisfaction and quality of life.

IMPLEMENTABILITY:
The Dutch Society of Obstetrics and Gynecology (NVOG) acknowledged the research question as an important knowledge gap, prioritized the project and agreed on adjustment of guidelines after proven (cost-)effectiveness. The strategy will be imbedded in the guideline

'Hevig menstrueel bloedverlies' and model protocol 'Submuceuze myomen, diagnostistiek and behandeling'. Beyond the scope of this research protocol, other gynaecological procedures (e.g. endometrial ablation) performed under general anesthesia for reasons of anxiety or pain perception can be performed under procedural sedation. This would lead to a further cost reduction besides offering a patient friendly treatment mode.

2. OBJECTIVES

Primary Objective: To demonstrate non-inferiority of complete hysteroscopic resection of submucous myomas under procedural sedation with propofol compared to hysteroscopic resection under general anesthesia.

Secondary Objective(s): To compare the cost effectiveness, pain, menstrual blood loss (PBAC score), quality of life, return to daily activities/work, hospitalization, (post)operative complications, re-interventions for hysteroscopic resection of submucous myomas under procedural sedation with propofol and for resection under general anesthesia.

Specific questions:

1. Does the use of procedural sedation for hysteroscopic myomectomy lead to a comparable same rate of complete resections, as compared to using general anesthesia?
2. What is the generic and specific quality of life measured with the Uterine Fibroid Symptom and Quality of Life questionnaire (UFS QoL) [26], EuroQoL (EQ-5D-5L) [27-28] and recovery index (RI-10) [29] after a hysteroscopic myomectomy using procedural sedation versus hysteroscopic myomectomy under general anesthesia?
3. What is the cost-effectiveness of hysteroscopic myomectomy using PSA versus hysteroscopic myomectomy under general anesthesia?

3. STUDY DESIGN

Study design: We aim to conduct a multicenter randomized non-inferiority trial comparing procedural sedation with general anesthesia in patients with symptomatic submucosal type 0 or I myomas up to 3 centimetres in diameter scheduled for hysteroscopic myomectomy. A cost-effectiveness study will be performed alongside the clinical study.

Setting: The proposed study will be performed within a consortium of university and teaching hospitals in the Netherlands. Patients will undergo hysteroscopic myomectomy either under general anesthesia or procedural sedation with propofol.

Duration: Preparation (6 months): protocol, database, approval medical-ethics committees. Inclusion (24 months): 205 patients. Follow-up (12 months): postoperative revision, recovery, complications. Analysis (6 months): data-analysis, publication.

4. STUDY POPULATION

4.1 Population (base)

We will study women with a minimum age of 18 years with a maximum of 2 symptomatic type 0 or I submucous myomas with a maximum diameter of 3 cm. They should be American Society of Anesthesiologist class 1 or 2 and able to sufficiently speak Dutch to fully understand the study and to complete the questionnaires.

4.2 Inclusion criteria

In order to be eligible to participate in this study, a subject must meet all of the following criteria:

- Minimum age: 18 years
- Symptomatic type 0 or I submucous myomas
- Maximum number of myomas: 2
- Maximum diameter of submucous myomas: 3 cm
- American Society of Anesthesiologist class 1 or 2

4.3 Exclusion criteria

A potential subject who meets any of the following criteria will be excluded from participation in this study:

- Aged under 18 years
- Inability to understand Dutch
- American Society of Anesthesiologist class 3 or 4
- Clotting disorders
- Severe anemia (Hb under 5.0 mmol/l).

4.4 Sample size calculation

The study is designed as a non-inferiority study, in which we want to investigate if hysteroscopic myomectomy under procedural sedation with propofol is non-inferior to the same surgical procedure under general anesthesia. With 205 women randomized we have 90% power to demonstrate non-inferiority, based on an estimated 2,5% incomplete resections in both groups, with an non-inferiority upper limit of 10% incomplete resections defined as non-inferior (i.e. a delta of 7.5%), an alpha of 0.025 and a drop-out rate of 10%.

5. TREATMENT OF SUBJECTS

5.1 Investigational product/treatment

Procedural sedation and analgesia in an outpatient setting

According to guidelines from the Health Care Inspectorate (IGZ) and Dutch Institute for Healthcare Improvement (CBO) non-anesthesiologist administered Propofol (NAAP) sedation is given and monitored by a qualified sedation practitioner [30-31]. The patient will be assessed by the sedation practitioner immediately prior to surgery on the basis of a pre-operative questionnaire. Non-invasive blood pressure, electrocardiogram and oxygen saturation are measured before vascular access is obtained. Propofol and alfentanil are used for procedural sedation.

Hysteroscopic resection is performed by an experienced surgeon by standard procedure in an office-based setting. Patients are observed after the procedure by qualified personnel and discharged as soon as all the discharge criteria are met, normally within 1 to 1.5 hours.

General anesthesia

General anesthesia can be volatile based or total intravenously, with the use of a laryngeal mask. Postoperatively, patients will be observed in the recovery room and discharged home from the clinic when all the discharge criteria are met.

The way hysteroscopic resection is performed under general anesthesia does not differ from the way it is performed under procedural sedation.

6. METHODS

6.1 Study parameters/endpoints

6.1.1 Main study parameter/endpoint

Primary outcome will be the percentage of complete resections, based on ultrasonography 6 weeks postoperatively by an independent gynaecologist or ultrasonographer blinded for the surgery outcome. This ultrasonography should be conducted in the follicular phase (first 14 days) of the menstrual cycle. A complete resection means that there are no signs of an intracavitary remaining of the myoma resected during hysteroscopic myomectomy on the transvaginal ultrasound.

6.1.2 Secondary study parameters/endpoints

Secondary outcomes are cost effectiveness, pain, menstrual blood loss (PBAC score), quality of life, return to daily activities/work, hospitalization, (post)operative complications, re-interventions. These secondary parameters will be assessed by several questionnaires.

6.2 Randomisation, blinding and treatment allocation

Randomisation will be performed web based through Alea with the use of a block design, with a variable block size. The expertise for this technology is already available for the study group (obs/gyn consortium). The study will not be double-blinded, as it is impossible to blind the health care workers and patients involved for the strategy to which the woman is allocated. The person performing the transvaginal ultrasonography at six weeks follow up, however, will be blinded for the study arm and judgement of completeness by the surgeon during the procedure. Patients will be able to complete the questionnaires online, through an link they receive by e-mail. In case patients wish to complete the questionnaires on paper, the research nurse will send the questionnaires by post at the follow-up moment.

6.3 Study procedures

Completeness of resection

The primary outcome parameter completeness of the resection will be evaluated by transvaginal ultrasonography (TVU) (contrast sonography if inconclusive TVU) by an independent gynecologist or ultrasonographer blinded for the treatment arm or judgment of completeness by the surgeon who performed the hysteroscopic myomectomy. This TVU should be performed 6 weeks postoperatively in the follicular phase of the menstrual cycle.

Baseline characteristics

At baseline the following characteristics are registered: body mass index, medical history, demographics, parity, age, smoking, type and number of myomas

Characteristics registered during the procedure

- completeness of resection as judged by surgeon
- surgical complications
- anesthesiologic complications (desaturation, airway obstruction, dysrhythmias, blood pressure drops, ECG alterations)
- operating time
- intravasation/fluid deficit
- recovery time (time from end of procedure until completely awake and communicative)
- use of pain medication and dose
- pain intensity measured by VAS scores
- duration of hospitalization (hours)

Characteristics registered after the procedure

Nausea and vomiting, sore throat, pain (VAS score)

Transvaginal Ultrasonography

Participating women will have had a TVU at baseline when the diagnosis of submucous myomas is made and the size and type of myomas is described. Another TVU follows at 6 weeks after hysteroscopic myomectomy to assess the completeness of resection. This TVU is part of the current usual care, so no extra ultrasonography is needed for this study.

Questionnaires

Participating women will fill in questionnaires at baseline, which will be given before the surgical procedure. During follow-up, patients will fill in questionnaires at 24 hours, 2 weeks, 8 weeks, 6 and 12 months after randomisation. Questionnaires will contain:

- Short questionnaire on side effects 24 hours after surgery.
- the EuroQoL (EQ-5D-5L) questionnaire (15 questions) to assess the quality of life
- PBAC scores to assess the amount of menstrual blood loss
- Uterine Fibroid Symptoms – Quality of Life (UFS-QoL) questionnaire (37 questions) to assess the quality of life focussing on patients suffering from uterine fibroids
- Recovery Index (RI-10) questionnaire (10 questions): to assess the patients' recovery process

- Medical Consumption Questionnaire (iMCQ)[32]: for cost effectiveness analysis
- Productivity Cost Questionnaire (iPCQ)[33] to assess productivity loss
- Short questionnaire on recurrence and re-interventions at 12 months after surgery.

	Side effects Pain (VAS)	EQ-5D-5L	PBAC	UFS-QoL	RI-10	iMCQ	iPCQ	Re-intervention
Baseline		X	X	X				
24 hours	X	X			X			
2 weeks		X			X			
8 weeks		X	X	X	X	X	X	
6 months						X		
12 months		X	X	X		X		X

A patient preference study (for which a different study protocol will be created) will be conducted alongside the trial including evaluation of patient satisfaction.

6.4 Withdrawal of individual subjects

Subjects can leave the study at any time for any reason if they wish to do so without any consequences. The investigator can decide to withdraw a subject from the study for urgent medical reasons.

7. SAFETY REPORTING

7.1 Section 10 WMO event

In accordance to section 10, subsection 1, of the WMO, the investigator will inform the subjects and the reviewing accredited METC if anything occurs, on the basis of which it appears that the disadvantages of participation may be significantly greater than was foreseen in the research proposal. The study will be suspended pending further review by the accredited METC, except insofar as suspension would jeopardise the subjects' health. The investigator will take care that all subjects are kept informed.

7.2 AEs, SAEs

7.2.1 Adverse events (AEs)

Adverse events are defined as any undesirable experience occurring to a subject during the study, whether or not considered related to [the investigational product / the experimental intervention]. All adverse events reported spontaneously by the subject or observed by the investigator or his staff will be recorded.

7.2.2 Serious adverse events (SAEs)

A serious adverse event is any untoward medical occurrence or effect that at any dose:

- results in death;
- is life threatening (at the time of the event);
- requires hospitalisation or prolongation of existing inpatients' hospitalisation;
- results in persistent or significant disability or incapacity;
- is a congenital anomaly or birth defect;
- Any other important medical event that may not result in death, be life threatening, or require hospitalization, may be considered a serious adverse experience when, based upon appropriate medical judgement, the event may jeopardize the subject or may require an intervention to prevent one of the outcomes listed above.

All SAEs will be reported by the local investigator to the sponsor as soon as possible, by using the form designed for this purpose, which will be available on the study website.,

The sponsor will report the SAEs through the web portal *ToetsingOnline* to the accredited METC that approved the protocol, within 15 days after the sponsor has first knowledge of the serious adverse events.

SAEs that result in death or are life threatening will be reported expedited. The expedited reporting will occur not later than 7 days after the responsible investigator has first knowledge of the adverse event. This is for a preliminary report with another 8 days for completion of the report.

7.3 Follow-up of adverse events

All AEs will be followed until they have abated, or until a stable situation has been reached. Depending on the event, follow up may require additional tests or medical procedures as indicated, and/or referral to the general physician or a medical specialist. SAEs need to be reported till end of study within the Netherlands, as defined in the protocol

7.4 Data Safety Monitoring Board (DSMB)

An independent Data Safety Monitoring Board (DSMB) will be asked to monitor the progress of the study and the safety of its participants. The DSMB will include: prof. dr. J.G.P. Tijssen, prof. dr. M.P.M. Burger, dr. J.H. van der Lee, dr. D.P. van der Ham and dr. T.R.de Haan. The DSMB will meet as required to review any expected adverse events and may ask to review outcomes or other data that may have an impact on the trial.

Given the low risk of serious adverse events, no interim analysis will be planned. Every 10 SAEs will be reported to the DSMB by the sponsor.

The advice of the DSMB will only be sent to the sponsor of the study. The sponsor will decide if the advice of the DSMB will be implemented. And will subsequently update the reviewing METC on this advice including a note to substantiate why (part of) the advice of the DSMB will not be followed).

8. STATISTICAL ANALYSIS

8.1 Data analysis and presentation/synthesis

Primary analysis will be by intention-to-treat. We will also perform a per protocol analysis, given the non-inferiority design of the study, where crossover between the groups will increase the changes of concluding non-inferiority, if in reality the treatment is not-inferior. We will present the percentage of complete resections at 6 weeks in both groups, with according relative risks and 95% confidence interval. Differences will be tested with the chi-square test, or, if the expected cell count is low using the Fisher exact test. We will also calculate the relative risk, adjusted for center where the procedure has been performed. Complications during and after surgery, and re-interventions will be categorized and evaluated by a Clinical Evaluation Committee, independent and blinded for the type of surgery performed (where possible). Average pain intensity during the procedure as well as the highest pain intensity during the procedure will be reported as means with SD, risk differences between both groups will be calculated with according 95% confidence interval. Time to recovery and pain will be visualized in a graph, and analyzed using a mixed model, that can take into account repeated measures in the same woman over time. The quality of life and PBAC scores will be analyzed according the developed algorithms.
We will perform a subgroup analysis for myoma size (<2 cm versus >=2 cm) and for parity (nulliparous versus multiparous women).

8.2 Cost effectiveness analysis (CEA)

GENERAL CONSIDERATIONS CEA

The aim of the economic evaluation is to relate the difference in societal and healthcare costs between procedural sedation and general anesthesia to the difference in clinical effects. Both a cost-effectiveness and cost-utility analysis will be performed with a time horizon of 12 months. Thus, discounting is not necessary.

COST ANALYSIS CEA

Costs will be measured from a societal perspective using internet questionnaires based on the iMCQ after 6 weeks, 3, 6 and 12 months of follow-up. Direct costs include costs of primary and secondary care, complementary care and home care. Indirect costs include absenteeism from paid and unpaid work, and presenteeism. The friction cost approach will be used to estimate indirect costs. For the valuation of health care utilization standard prices published in the Dutch costing guidelines [34] will be used.
Medication use will be valued using prices of the Royal Dutch Society for Pharmacy [35].

PATIENT OUTCOME ANALYSIS CEA

Societal costs will be related to the following effect measures in the economic evaluation:

1) % of complete resection;

2) quality-adjusted life-years (QALYs) based on the Dutch tariff for the EuroQol (EQ-5D-5L) [36-37].

The analysis will be done according to the intention-to-treat principle. Missing cost and effect data will be imputed using multiple imputation. Incremental cost-effectiveness ratios (ICERs) will be calculated by dividing the difference in mean total costs between the treatment groups by the difference in mean effects. Bootstrapping with 5000 replications will be used to estimate 95% confidence intervals around cost differences and the uncertainty surrounding the ICERs. Uncertainty surrounding the ICERs will be graphically presented on cost-effectiveness planes. Cost-effectiveness acceptability curves showing the probability that the intervention is cost-effective in comparison with usual care for a range of different ceiling ratios will also be estimated [38]. Adjustment for confounders and effect modifiers will be done if necessary.

8.3 Budget Impact Analysis (BIA)

GENERAL CONSIDERATIONS BIA

The budget impact analysis will be performed based on the recommendations from Sullivan et al [39]. In the budget impact analysis, the effectiveness of the treatments will be extrapolated over a period of 5 years based on the estimates obtained from the proposed study. Perspectives that will be considered are the societal, government (Budget Kader Zorg) and insurer perspective. Different implementation scenarios (ranging from 0% to 100% implementation) will be evaluated.

COST ANALYSIS BIA

The total number of patients eligible for the intervention will be estimated based on Dutch epidemiological data. Resource utilization is calculated by multiplying the number of eligible patients with the resource utilization rates obtained from the economic evaluation. Different prices will be used to value resource use depending on the perspective of the analysis: Dutch standard costs for the societal perspective, actual NZA tariffs for the government perspective, and average tariffs NZA for the insurer perspective. Both resource use and annual costs will be presented over a 5 year period for all perspectives. Aggregated and disaggregated (e.g. GP care, secondary care, and productivity losses) total costs per year will be presented for the different perspectives and scenarios.

8.4 Interim analysis

Given the low risk of adverse events, an interim analysis will not be planned.

9. ETHICAL CONSIDERATIONS

9.1 Regulation statement

The study will be conducted according to the principles of the Declaration of Helsinki (WORLD MEDICAL ASSOCIATION DECLARATION OF HELSINKI Ethical Principles for Medical Research Involving Human Subjects, Version Fortaleza, Brazil, October 2013,) and in accordance with the Medical Research Involving Human Subjects Act (WMO).

9.2 Recruitment and consent

The gynaecologist participating in the network will inform the patient about the study and refer the patients to dedicated research nurses. These nurses will counsel patients, ask for informed consent, and perform randomisation. The research nurse will organize the required treatments, depending on the result of the randomisation.

9.3 Benefits and risks assessment, group relatedness

This study will not impose extra risk on women participating in the trial, since it compares anesthetic procedures that are all used in daily practice. Hysteroscopic myomectomy is performed in a similar way regardless of the type of anesthesia that is applied. Hysteroscopic myomectomy using procedural sedation is expected to be equally effective as under general anesthesia. A significant cost reduction is expected, whilst patient satisfaction/preference is comparable or increased. Therefore research in order to optimize treatment is necessary.

9.4 Compensation for injury

The Máxima Medical Centre has an insurance which is in accordance with the legal requirements in the Netherlands (Article 7 WMO). This insurance provides cover for damage to research subjects through injury or death caused by the study.
The insurance applies to the damage that becomes apparent during the study or within 4 years after the end of the study.

10. ADMINISTRATIVE ASPECTS, MONITORING AND PUBLICATION

10.1 Handling and storage of data and documents

Data will be collected using a website dedicated to studies in the Dutch Consortium for women's health and reproductivity studies (www.studies-obsgyn.nl). Similar website oriented databases have already been developed.
Data monitoring will be done by research nurses in each of the participating centres. A substantial part of these research nurses is already working for the above mentioned consortium. Participants will be given a computer generated numeric code. Data handling will be done anonymously, with the patient code only available to the local investigator and the research nurse working in the local centre.
In accordance with guidelines of the Dutch Federation of University Medical Centers (NFU) the data will be kept for 15 years.

10.2 Amendments

Amendments are changes made to the research after a favourable opinion by the accredited METC has been given. All amendments will be notified to the METC that gave a favourable opinion.
Non-substantial amendments will not be notified to the accredited METC and the competent authority, but will be recorded and filed by the sponsor.

10.3 Annual progress report

The sponsor/investigator will submit a summary of the progress of the trial to the accredited METC once a year. Information will be provided on the date of inclusion of the first subject, numbers of subjects included and numbers of subjects that have completed the trial, serious adverse events/ serious adverse reactions, other problems, and amendments.

10.4 End of study report

The investigator will notify the accredited METC of the end of the study within a period of 8 weeks. The end of the study is defined as the last patient's last visit.
In case the study is ended prematurely, the investigator will notify the accredited METC within 15 days, including the reasons for the premature termination.
Within one year after the end of the study, the investigator/sponsor will submit a final study report with the results of the study, including any publications/abstracts of the study,

to the accredited METC.

10.5 Public disclosure and publication policy

The principal investigator will publish the results of the study in a peer reviewed medical journal as soon as appropriate.

11. REFERENCES

1. Varma R, Soneja H, Clark TJ, Gupta JK. Hysteroscopic myomectomy for menorrhagia using Versascope bipolar system: efficacy and prognostic factors at a minimum of one year follow up. Eur J Obstet Gynecol Reprod Biol. 2009 Feb;142(2):154-9.
2. Vercellini P, Zàina B, Yaylayan L, Pisacreta A, De Giorgi O, Crosignani PG. Hysteroscopic myomectomy: long-term effects on menstrual pattern and fertility. Obstet Gynecol. 1999 Sep;94(3):341-7.
3. Emanuel MH, Hart A, Wamsteker K, Lammes F. An analysis of fluid loss during transcervical resection of submucous myomas. Fertil Steril. 1997 Nov;68(5):881-6.
4. Hart R, Molnár BG, Magos A. Long term follow up of hysteroscopic myomectomy assessed by survival analysis. Br J Obstet Gynaecol. 1999 Jul;106(7):700-5.
5. Polena V, Mergui JL, Perrot N, Poncelet C, Barranger E, Uzan S. Long-term results of hysteroscopic myomectomy in 235 patients. Eur J Obstet Gynecol Reprod Biol. 2007 Feb;130(2):232-7.
6. Di Spiezio Sardo A, Mazzon I, Bramante S, Bettocchi S, Bifulco G, Guida M, Nappi C. Hysteroscopic myomectomy: a comprehensive review of surgical techniques. Hum Reprod Update. 2008 Mar-Apr;14(2):101-19.
7. Marsh F, Kremer C, Duffy S. Delivering an effective outpatient service in gynaecology. A randomised controlled trial analysing the cost of outpatient versus daycase hysteroscopy. BJOG. 2004 Mar;111(3):243-8.
8. Timmermans A, van Dongen H, Mol BW, Veersema S, Jansen FW. Hysteroscopy and removal of endometrial polyps: a Dutch survey. Eur J Obstet Gynecol Reprod Biol. 2008 May;138(1):76-9.
9. Bettocchi S, Ceci O, Nappi L, Di Venere R, Masciopinto V, Pansini V, Pinto L, Santoro A, Cormio G. Operative office hysteroscopy without anesthesia: analysis of 4863 cases performed with mechanical instruments. J Am Assoc Gynecol Laparosc. 2004 Feb;11(1):59-61.
10. Pakrashi T. New hysteroscopic techniques for submucosal uterine fibroids. Curr Opin Obstet Gynecol. 2014 Aug;26(4):308-13
11. Garewal D, Powell S, Milan SJ, Nordmeyer J, Waikar P. Sedative techniques for endoscopic retrograde cholangiopancreatography. Cochrane Database Syst Rev. 2012 Jun 13;6:CD007274
12. Singh H, Poluha W, Cheung M, Choptain N, Baron KI, Taback SP. Propofol for sedation during colonoscopy. Cochrane Database Syst Rev. 2008 Oct 8;(4):CD006268
13. Metzner J, Domino KB. Risks of anesthesia or sedation outside the operating room: the role of the anesthesia care provider. Curr Opin Anesthes 2010

14. Cravero JP, Beach ML, Blike GT, Gallagher SM, Hertzog JH. The incidence and nature of adverse events during pediatric sedation/anesthesia with propofol for procedures outside the operating room: a report from the Pediatric Sedation Research Consortium. Anesth Analg. 2009 Mar;108(3):795-804

15. Karamnov S, Sarkisian N, Grammer R, Gross WL, Urman RD. Analysis of Adverse Events Associated With Adult Moderate Procedural Sedation Outside the Operating Room. J Patient Saf. 2014 Sep 8.

16. Di Spiezio Sardo A, Taylor A, Tsirkas P, Mastrogamvrakis G, Sharma M, Magos A. Hysteroscopy: a technique for all? Analysis of 5,000 outpatient hysteroscopies. Fertil Steril. 2008 Feb;89(2):438-43.

17. Wortman M, Daggett A, Ball C. Operative hysteroscopy in an office-based surgical setting: review of patient safety and satisfaction in 414 cases. J Minim Invasive Gynecol. 2013 Jan-Feb;20(1): 56-63

18. Di Spiezio Sardo A, Bettocchi S, Spinelli M, Guida M, Nappi L, Angioni S, Sosa Fernandez LM, Nappi C. Review of new office-based hysteroscopic procedures 2003-2009. J Minim Invasive Gynecol. 2010 Jul-Aug;17(4):436-48

19. Richtlijn Hevig Menstrueel Bloedverlies (NVOG) (http://nvogdocumenten.nl/index.php?pagina=/richtlijn/pagina.php&fSelectTG_62=75&fSelectedSub=62&fSelectedParent=75)

20. McQuaid KR, Laine L. A systematic review and meta-analysis of randomized, controlled trials of moderate sedation for routine endoscopic procedures. Gastrointest Endosc. 2008 May;67(6):910-23

21. Clark TJ, Samuel N, Malick S, Middleton LJ, Daniels J, Gupta JK. Bipolar radiofrequency compared with thermal balloon endometrial ablation in the office: a randomized controlled trial. Obstet Gynecol. 2011 Jan;117(1):109-18.

22. Smith PP, Middleton LJ, Connor M, Clark TJ. Hysteroscopic morcellation compared with electrical resection of endometrial polyps: a randomized controlled trial. Obstet Gynecol. 2014 Apr;123(4):745-51

23. Nilsson A, Nilsson L, Ustaal E, Sjöberg F. Alfentanil and patient-controlled propofol sedation – facilitate gynaecological outpatient surgery with increased risk of respiratory events. Acta Anaesthesiol Scand 2012; 56(9): 1123-9.

24. Lok IH, Chan M, Tam WH, Leung PL, Yuen PM. Patient-controlled sedation for outpatient thermal balloon endometrial ablation. J Am Assoc Gynecol Laparosc. 2002 Nov;9(4):436-41.

25. Lasmar RB, Xinmei Z, Indman PD, Celeste RK, Di Spiezio Sardo A. Feasibility of a new system of classification of submucous myomas: a multicenter study. Fertil Steril. 2011 May;95(6):2073-7

26. Spies JB, Coyne K, Guaou Guaou N, Boyle D, Skyrnarz-Murphy K, Gonzalves SM. The UFS-QOL, a new disease-specific symptom and health-related quality of life questionnaire for leiomyomata. Obstet Gynecol. 2002 Feb;99(2):290-300.
27. www.euroqol.org
28. Janssen MF, Pickard AS, Golicki D, Gudex C, Niewada M, Scalone L, Swinburn P, Busschbach J. Measurement properties of the EQ-5D-5L compared to the EQ-5D-3L across eight patient groups: a multi-country study. Qual Life Res. 2013; 22(7): 1717–1727. 29. Kluivers KB, Hendriks JC, Mol BW, Bongers MY, Vierhout ME, Brölmann HA, de Vet HC. Clinimetric properties of 3 instruments measuring postoperative recovery in a gynecologic surgical population. Surgery. 2008 Jul;144(1):12-21.
30. Richtlijn Sedatie en/of analgesie (PSA) op locaties buiten de operatiekamer. Kwaliteitsinstituut voor de Gezondheidszorg CBO, 2009
31. Toetsingskader sedatie en/of analgesie buiten de operatiekamer. Inspectie voor de Gezondheidszorg, 2012
32. www.imta.nl (C. Bouwmans, L. Hakkaart-van Roijen, M. Koopmanschap, M. Krol, H.Severens, W. Brouwer.Handleiding iMTA Medical Cost Questionnaire (iMCQ). Rotterdam: iMTA, Erasmus Universiteit Rotterdam, 2013)
33. www.imta.nl (C. Bouwmans, L. Hakkaart-van Roijen, M. Koopmanschap, M. Krol, H.Severens, W. Brouwer.Handleiding iMTA Productivity Cost Questionnaire (iPCQ). Rotterdam: iMTA, Erasmus Universiteit, 2013)
34. Hakkaart-van Roijen L, Tan SS, Bouwmans CAM. Handleiding voor kostenonderzoek: Methoden en standaard kostprijzen voor economische evaluaties in de gezondheidszorg. Geactualiseerde versie 2010. [Dutch manual for costing in economic evaluations]. Diemen: College voor zorgverzekeringen (CVZ)2011.
35. Z-index. G-Standaard. The Hague, The Netherlands: Z-index 2006
36. EuroQol Group. EuroQol--a new facility for the measurement of health-related quality of life. The EuroQol Group. Health Policy. 1990;16(3):199-208.
37. Lamers LM, Stalmeier PF, McDonnell J, Krabbe PF, van Busschbach JJ. [Measuring the quality of life in economic evaluations: the Dutch EQ-5D tariff]. Ned Tijdschr Geneeskd. 2005;149(28):1574-8.
38. Fenwick E, O'Brien BJ, Briggs A. Cost-effectiveness acceptability curves--facts, fallacies and frequently asked questions. Health Econ. 2004;13(5):405-15
39. Sullivan SD, Mauskopf JA, Augustovski F, Jaime Caro J, Lee KM, Minchin M, Orlewska E, Penna P, Rodriguez Barrios JM, Shau WY. Budget impact analysis-principles of good practice: report of the ISPOR 2012 Budget Impact Analysis Good Practice II Task Force. Value Health. 2014 Jan-Feb;17(1):5-14
