# Supplementary material for: Procedural sedation and analgesia versus general anesthesia for hysteroscopic myomectomy (PROSECCO trial): A multicenter randomized controlled trial
Source: PLoS Med. 2023 Dec 28;20(12):e1004323. doi: 10.1371/journal.pmed.1004323 (PMC10754450; doi:10.1371/journal.pmed.1004323)
Supplement: S1 Text — Appendix 1. Questionnaires. Vragenlijst bijwerkingen 24 uur na hysteroscopische myoomresectie (Dutch version). Questionnaire on side effects 24 hours after hysteroscopic myomectomy (English version). Vragenlijst evaluatie recidief na hysteroscopische myoomresectie (Dutch version). Questionnaire for evaluation of recurrence after hysteroscopic myomectomy (English version). Appendix 2. Supplementary tables. Table A. Surgical and anesthesia details. Table B. Subgroup analyses for primary outcome. Table C. Surgeon’s judgment on completeness and ease of procedure. Table D. NRS score. Table E. Recovery Index Questionnaire. Table F. Surgical complications. Table G. Postoperative complications. Table H. PBAC score. Table I. EQ-5D-5L. Table J. UFS-QoL. Appendix 3. Statistical Analysis Plan. (PDF) [file pmed.1004323.s003.pdf]

Supporting information PROSECCO trial

Table of Contents

Appendix 1 – Questionnaires 2
Appendix 2 – Supplementary tables 6
Appendix 3 – Statistical Analysis Plan PROSECCO trial 13

Appendix 1. Questionnaires

Vragenlijst bijwerkingen 24 uur na hysteroscopische myoomresectie (Dutch version)

1. Datum van invullen: ....(dd/mm/jaar)

2. Hoe misselijk bent u op dit moment?

Omcirkel hieronder alstublieft het getal dat het beste uw mate van misselijkheid op dit moment aangeeft. Een '0' betekent niet misselijk, een '10' betekent de meest denkbare misselijkheid.

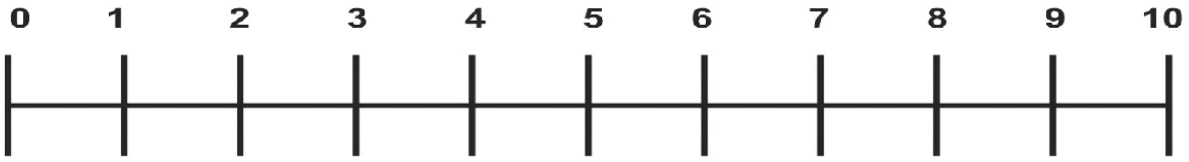

Niet misselijk

Meest denkbare misselijkheid

3. Hebt u moeten overgeven na de operatie?

Ja □

Nee □

Indien u de vorige vraag met ja hebt beantwoord:

4. Hoe vaak hebt u na de operatie moeten overgeven? ………………….. keer

5. Hoeveel pijn hebt u op dit moment?

Omcirkel hieronder alstublieft het getal dat het beste uw pijn op dit moment aangeeft. Een '0' betekent geen pijn, een '10' betekent de meest denkbare pijn.

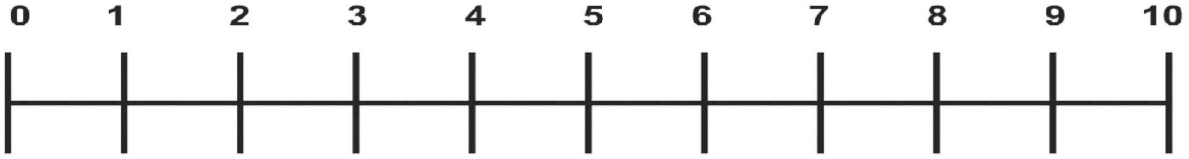

Geen pijn

Meest denkbare pijn

Questionnaire on side effects 24 hours after hysteroscopic myomectomy (English Version)

1. Date while filling in this questionnaire: ....(day/month/year)

2. How nauseous are you at the moment?

At this scale, please mark the number that indicates how nauseous you feel at the moment. ‘0’ means you don’t experience any nausea, ’10’ means the most extreme nausea you can imagine.

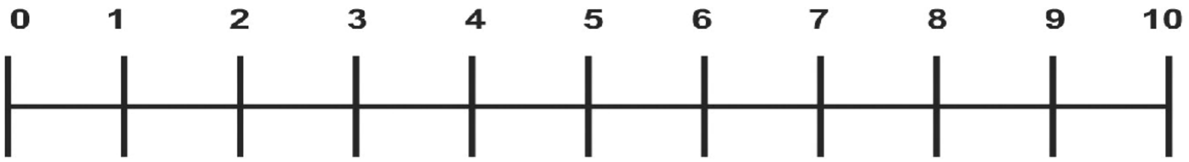

No nausea

Most extreme nausea

3. Did you have to throw up after the surgery?
   - Yes □
   - No □

Did you check yes? Go to question 4. Otherwise skip to question 5.

4. How many times did you have to throw up after the surgery?…………………..times

5. How much pain do you experience at the moment?

At this scale, please mark the number that indicates how much pain you experience at the moment. ‘0’ means you don’t experience any pain, ’10’ means the most extreme pain you can imagine.

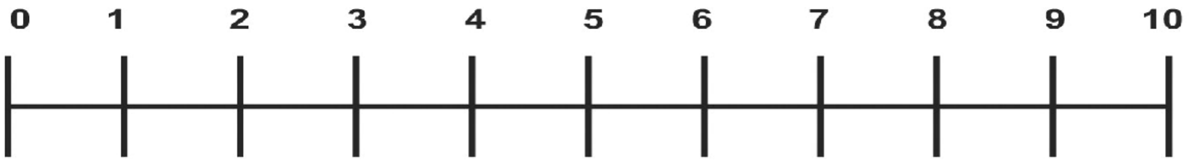

No pain

Worst pain you can imagine

Vragenlijst evaluatie recidief na hysteroscopische myoomresectie (Dutch version)

Het is nu een jaar geleden dat u de hysteroscopische myoomresectie heeft ondergaan (het verwijderen van de vleesboom met een kijkbuisje via de vagina). Wij zijn benieuwd of deze vleesboom het afgelopen jaar is teruggekomen en of u hiervoor een behandeling hebt ondergaan. Geeft u daarom zo nauwkeurig mogelijk antwoord op onderstaande vragen, door het juiste hokje aan te vinken.

1. Datum van invullen: ....(dd/mm/jaar)

2. Is de vleesboom waarvoor u behandeld bent het afgelopen jaar teruggekomen?

   Ja ☐

   Nee ☐

Indien u de vorige vraag met ja hebt beantwoord, ga dan verder met vraag 3.
Indien u de vorige vraag met nee hebt behandeld, dan is dit het einde van de vragenlijst.

3. Kreeg u opnieuw klachten door het terugkomen van de vleesboom?

   Ja ☐

   Nee ☐

Indien u de vorige vraag met ja hebt beantwoord:

4. Hebt u hiervoor een behandeling ondergaan?

   Ja ☐

   Nee ☐

Indien u de vorige vraag met ja hebt beantwoord:

5. Welke behandeling hebt u ondergaan?

☐ Gestart met medicatie vanwege uw menstruatiecyclus? (de pil, MIRENA spiraaltje, cyklokapron)
Datum: …………..……………..

☐ Het verwijderen van de vleesboom met een kijkbuisje via de vagina (hysteroscopische myoomresectie)
Datum: …………..……………..

☐ Het wegbranden van het baarmoederslijmvlies (endometrium ablatie)
Datum: …………..……………..

☐ Het verwijderen van de baarmoeder

Op welke manier is de baarmoeder verwijderd?

- ☐ via de vagina
- ☐ via de buik
- ☐ met kijkbuisjes (laparoscopisch)

Datum: …………..……………..

Einde vragenlijst

Questionnaire for evaluation of recurrence after hysteroscopic myomectomy (English version)

It has been a year since you underwent the hysteroscopic myomectomy (the removal of a fibroid with a small instrument through the vagina).
We would like to know if you experienced a recurrence of your fibroid and whether or not you underwent treatment for this during the last year.
To evaluate this, please answer the following questions by checking the appropriate box.

1. Date while filling in this questionnaire: ....(day/month/year)

2. Did the fibroid, that you were operated for, return in the last year?
   Yes □
   No □

Did you check yes? Go to question 3. Otherwise, this is the end of the questionnaire.

3. Did you experience new complaints because the fibroid did return?
   Yes □
   No □

Did you check yes? Go to question 4. Otherwise, this is the end of the questionnaire.

4. Did you receive treatment for this?
   Yes □
   No □

Did you check yes? Go to question 5. Otherwise, this is the end of the questionnaire.

5. Which treatment did you receive?

□ Start with medication because of your menstrual cycle? (oral contraception, MIRENA iud, cyklokapron)
Date: …………..……………..

□ Removal of the fibroid with a small instrument through the vagina (hysteroscopic myomectomy)
Date: …………..……………..

□ Operation to remove or thin the endometrium (the lining of the uterus (womb): endometrial ablation
Date: …………..……………..

□ Removal of the uterus (womb)
- □ through the vagina: vaginal hysterectomy
- □ through a cut in your tommy: abdominal hysterectomy
- □ by keyhole surgery: laparoscopic hysterectomy

Date: …………..……………..

End of the questionnaire

Appendix 2 – Supplementary tables

Table A. Surgical and anesthesia details

Variable	PSA N=106	GA N=101	Rel. risk (95% CI)	p-value
Anesthesia not according to randomisation:	5 (4.7%)	2 (2.0%)	2.38 (0.47 – 12.0)	0.45 *
Reason:				
patient's own wish	2 / 5 (40.0%)	2 / 2 (100%)		
anesthesiologist's decision	1 / 5 (20.0%)	0 / 2 (0.0%)		
unknown	2 / 5 (40.0%)	0 / 2 (0.0%)		
Analgesics during procedural sedation				
alfentanil	72 (72.0%)	n/a	n/a	-
remifentanil	28 (28.0%)	n/a	n/a	-
Analgesics used prophylactically	70 (70.0%)	63 (68.5%)	1.02 (0.85 – 1.24)	0.82
paracetamol	20 / 70 (28.6%)	24 / 63 (38.1%)		
NSAIDs	10 / 70 (14.3%)	2 / 63 (3.2%)		
paracetamol and NSAID	34 / 70 (48.6%)	33 / 63 (52.4%)		
paracetamol, NSAID and opiate	3 / 70 (4.3%)	3 / 63 (4.8%)		
paracetamol and opiate	3 / 70 (4.3%)	1 / 63 (1.6%)		
Procedure timed in first half of menstrual cycle	23 (30.3%)	27 (42.9%)	0.71 (0.45 – 1.10)	0.12
Resection technique				0.05
resectoscope	57 (53.8%)	67 (67.0%)		
morcellation	49 (46.2%)	33 (33.0%)		
Fluid loss, mL (median, IQR)	700 (257.5 – 1550)	600 (200 – 1335)	98.5 (-60 – 255) †	0.23
Time in surgery, minutes (median, IQR)	24.5 (19.0 – 37.0)	25.0 (17.0 – 32.0)	2.0 (-2.0 – 5.0) †	0.28
Presence of intracavitary polyp	18 (16.9%)	14 (13.9%)	1.22 (0.64 – 2.33)	0.54
removed during procedure	17 / 18 (94.4%)	14 / 14 (100.0%)		

Supplementary table A | Surgical and anesthesia details. IQR: Interquartile Range. Percentages are column percentages based on the number of observations available (i.e. excluding missing observations). Relative risks with 95% confidence intervals and Chi-squared test. * Fisher's exact test. † Difference in median with Hodges-Lehmann estimator and p-value for Mann-Whitney test.

Table B. Subgroup analyses for primary outcome

Variable	Intention-to-treat PSA N=98	Intention-to-treat GA N=89	Intention-to-treat Risk diff. (95%CI)	Intention-to-treat P-value interaction	Per protocol PSA N=94	Per protocol GA N=85	Per protocol Risk diff. (95%CI)	Per protocol P-value interaction
Parity								
nulliparous	29 (82.9%)	32 (97.0%)	-14.11% (-27.45 - -0.77)	0.0287	29 (85.2%)	32 (97.0%)	-11.68% (-24.49 – 1.14)	0.05
primi- and multiparous	54 (90.0%)	45 (83.3%)	6.67% (-6.13 – 19.47)		51 (89.5%)	43 (82.7%)	6.78% (-6.53 – 20.09)	
Myoma size								
< 20 mm	33 (89.2%)	45 (93.8%)	-4.56% (-17.16 – 8.04)	0.37	33 (89.2%)	43 (93.5%)	-4.29% (-17.06 – 8.48)	0.31
≥ 20 mm	53 (86.9%)	34 (82.9%)	3.96% (-10.66 – 18.58)		50 (87.7%)	34 (82.9%)	4.79% (-9.76 – 19.35)	

Supplementary table B | Subgroup analyses. PSA: Procedural sedation and analgesia, GA: general anesthesia, CI: confidence interval. Results of subgroup analyses are subject to confounding and multiple testing and should be interpreted with caution. P-values for interaction of treatment effect and sub-group.

Table C. Surgeon's judgement on completeness and ease of procedure

Variable	PSA N=106	GA N=101	Rel. risk (95% CI)	p-value
Resection judged complete by surgeon	79 (74.5%)	82 (82.8%)	0.90 (0.78 – 1.04)	0.15
Reason when incomplete:*				
intramural extension of myoma	10 / 27 (37.0%)	6 / 17 (35.3%)		
amount of fluid loss	15 / 27 (55.6%)	8 / 17 (47.1%)		
complication	1 / 27 (3.7%)	2 / 17 (11.8%)		
poor visualization	7 / 27 (25.9%)	6 / 17 (35.3%)		
patient discomfort / pain	0 / 27 (0.0%)	0 / 17 (0.0%)		
patient movement	1 / 27 (3.7%)	0 / 17 (0.0%)		
technical problems	4 / 27 (14.8%)	4 / 17 (23.5%)		
other	3 / 27 (11.1%)	4 / 17 (23.5%)		
Surgeon's judgement on ease of procedure:				
easy no limiting factors	61 (58.7%)	62 (63.3%)	0.93 (0.74 – 1.16)	0.50
view/access limited, performance not influenced	19 (18.3%)	23 (23.5%)		
view/access limited, influencing performance	21 (20.2%)	10 (10.2%)		
not possible to perform procedure	3 (2.9%)	3 (3.1%)		
When incomplete, 2nd session	12/27 (44.4%)	7/17 (41.2%)		
complete	9 (75.0%)	5 (83.3%)		
When incomplete, need for 3rd session	0 (0.0%)	0 (0.0%)		

Supplementary table C | Surgeon's judgement on completeness and ease of procedure. PSA: Procedural sedation and analgesia, GA: General anesthesia. Percentages are column percentages based on the number of observations available (i.e. excluding missing observations).*multiple answers can apply.

Table D. NRS score

NRS score	Recovery room	Discharge	24 hours
PSA	2.0 (0.0 – 4.0) N=91	2.0 (0.0 – 2.0) N=79	2.5 (1.0 – 5.0) N=90
GA	0.0 (0.0 – 2.0) N=91	1.0 (0.0 – 2.0) N=91	2.0 (1.0 – 4.0) N=72
Difference between groups	*0.0 (0.00 – 1.00) P=0.027*	0.0 (0.00 – 1.00) *P=0.319*	0.0 (0.00 – 1.00) *P=0.256*

Supplementary table D | NRS score at return in recovery room, discharge and 24 hours follow-up. NRS: Numeric Rating Scale. PSA: Procedural sedation and analgesia, GA: General anesthesia. Medians with interquartile ranges. Hodges-Lehmann estimator for difference in median between groups with p-value for Mann-Whitney test.

Table E. Recovery Index Questionnaire

RI10 score	24 hours	2 weeks	8 weeks
PSA	35.0 (27.0 – 40.0)<br>N=89	40.0 (34.0 – 45.5)<br>N=87	45.0 (38.0 – 49.0)<br>N=76
GA	32.0 (26.5 – 39.5)<br>N=72	39.0 (33.0 – 44.0)<br>N=68	44.0 (37.0 – 47.0)<br>N=66
Difference between groups	1.0 (-2.0 – 4.0)<br>*P=0.40*	1.0 (-1.0 – 4.0)<br>*P=0.40*	1.0 (-1.0 – 3.0)<br>*P=0.25*

Supplementary table E | RI10 score at 24 hours, 2 weeks and 8 weeks follow-up. RI10: Recovery Index Questionnaire. PSA: Procedural sedation and analgesia, GA: General anesthesia. Medians with interquartile ranges. Hodges-Lehmann estimator for difference in median between groups with p-value for Mann-Whitney test.

Table F. Surgical complications

Variable	PSA<br>N=106	GA<br>N=101	Rel. risk (95% CI)	p-value
Surgical complications	8 (7.5%)	5 (5.0%)	1.53 (0.52 – 4.51)	0.44
perforation of uterine wall	1 / 8 (12.5%)	3 / 5 (60.0%)		
extravasation resulting in electrolyte disturbances	1/ 8 (12.5%)	1 / 5 (20.0%)		
haemorrhage	3 / 8 (37.5%)	0 / 7 (0.0%)		
cervical laceration	3 / 8 (37.5%)	1 / 5 (20.0%)		
Treatment of complication	4 (50.0%)	2 (40.0%)		
medication given	2 / 4	2 / 4		
antibiotics	0 / 2	1 / 2		
diuretics	1 / 2	1 / 2		
tranexamic acid	1 / 2	0 / 2		
other	2 / 4	0 / 4		

Supplementary table F | Surgical complications. PSA: Procedural sedation and analgesia, GA: General anesthesia. Percentages are column percentages based on the number of observations available (i.e. excluding missing observations). Relative risk with 95% confidence interval and Chi-squared test.

Table G. Postoperative complications

Variable	PSA N=106	GA N=101	Rel. risk (95% CI)	P-value
Complications between discharge and follow-up visit	9 (8.5%)	6 (6.1%)	1.40 (0.52 – 3.79)	0.50
If complication, specification*				
infection	2	0		
haemorrhage	5	0		
pain	3	4		
other	1	2		
Treatment of complication*	7 / 9 (77.8%)	3 / 6 (50.0%)		
medication	5	1		
need for new appointment	3	1		
need for re-operation	1	0		
other	0	1		

Supplementary table G | Postoperative complications until 6 weeks follow-up. PSA: Procedural sedation and analgesia, GA: General anesthesia. Percentages are column percentages based on the number of observations available (i.e. excluding missing observations). Relative risk with 95% confidence interval and Chi-squared test. * multiple answers can apply.

Table H. PBAC score

PBAC-score	Baseline	8 weeks	12 months	Overall*
PSA	172.0 (45.0 – 370.0)<br>N=69	72.0 (7.0 – 196.0)<br>N=69	5.0 (0.0 – 62.0)<br>N=55	
Change within group		-81.5 (-308.0 – 0.0) *P= <0.0001*	-120 (-331 – -19.0) *P =<0.0001*	
GA	192.0 (70.5 – 376.5)<br>N=56	22.0 (0.0 – 99.0)<br>N=49	19.0 (0.0 – 128.0)<br>N=42	
Change within group		-153.0 (-320.0 – -1.0) *P=<0.0001*	-102.0 (-320.0 – 6.0) *P=0.04*	
Difference between groups		*41.0 (-37.0 – 148.0)*<br>*P=0.30*	*-35.0 (-127.0 – 60.0)*<br>*P=0.38*	*-16.0 (-54.0 – -22.0)*<br>*P=0.41*

Supplementary table H | PBAC score at baseline, 8 weeks, 12 months follow-up. Medians and interquartile ranges. PBAC: Pictorial Blood Assessment Chart, PSA: Procedural sedation and analgesia, GA: General anesthesia. Sign test for within group change from baseline. Difference in median difference from baseline between groups with Hodges-Lehman estimator with p-value for Mann-Whitney test. *Overall estimate is mean difference over all time points with a Poisson distribution using a GEE repeated measures model including variables adjusting for baseline score, time and interaction of treatment effect and time.

Table I. EQ-5D-5L

EQ-5D-5<br>Index score	Baseline	24 hours	2 weeks	8 weeks	6 months	12 months
PSA	0.87 (0.73 – 1.00)<br>N=90	0.84 (0.78 – 0.87)<br>N=90	0.87 (0.84 – 1.00)<br>N=87	1.00 (1.00 – 1.00)<br>N=77	1.00 (0.87 – 1.00)<br>N=76	1.00 (1.00 – 1.00)<br>N=68
Change within group		0.00 (-0.13 – 0.04)<br>*P=0.28*	0.00 (-0.00 – 0.13)<br>*P=0.064*	0.06 (0.00 – 0.16)<br>*P=<0.0001*	0.00 (0.00 – 0.16)<br>*P=0.009*	0.07 (0.00 – 0.19)<br>*P=<0.0001*
GA	0.84 (0.79 – 1.00)<br>N=78	0.83 (0.73 – 0.87)<br>N=71	0.87 (0.84 – 1.00)<br>N=69	1.00 (0.87 – 1.00)<br>N=68	1.00 (0.85 – 1.00)<br>N=61	1.00 (0.94 – 1.00)<br>N=55
Change within group		-0.03 (-0.17 – 0.05)<br>*P=0.049*	0.00 (-0.04 – 0.16)<br>*P=0.40*	0.04 (0.00 – 0.16)<br>*P=<0.0001*	0.03 (0.00 – 0.16)<br>*P=0.003*	0.07 (0.00 – 0.16)<br>*P=0.001*
Difference between groups		0.00 (-0.03 – 0.04)<br>*P=0.64*	0.00 (0.00 – 0.00)<br>*P=0.78*	0.00 (0.00 – 0.00)<br>*P=0.18*	0.00 (0.00 – 0.00)<br>*P=0.29*	0.00 (0.00 – 0.00)<br>*P=0.30*

EQ-5D-5L VAS score	Baseline	24 hours	2 weeks	8 weeks	6 months	12 months
PSA	80.0 (70.0 – 90.0)<br>N=90	75.0 (60.0 – 86.0)<br>N=90	80.0 (72.5 – 90.0)<br>N=87	85.0 (80.0 – 90.0)<br>N=77	85.0 (80.0 – 90.0)<br>N=76	85.0 (80.0 – 90.0)<br>N=68
Change within group		-5 (-19.0 – 5.0)<br>*P=0.040*	0.0 (-5.0 – 10.0)<br>*P=0.34*	5.0 (0.0 – 15.0)<br>*P=<0.0001*	5 (-4.5 – 14.0)<br>*P=0.016*	5.0 (0.0 – 10.0)<br>*P= <0.0001*
GA	80.0 (70.0 – 90.0)<br>N=78	70.0 (60.0 – 80.0)<br>N=72	80.0 (70.0 – 90.0)<br>N=69	85.0 (75.5 – 90.0)<br>N=68	85.0 (75.0 – 90.0)<br>N=61	85 (80.0 – 95.0)<br>N=55
Change within group		-10.0 (-19.5 – 0.0)<br>*P=<0.0001*	0.0 (-5.0 – 5.0)<br>*P=1.00*	0.0 (0.0 – 10.0)<br>*P=0.017*	5.0 (0.0 – 11.5)<br>*P=0.009*	5.0 (-2.5 – 15.0)<br>*P=0.005*
Difference between groups		1.00 (-5.0 – 10.0)<br>*P=0.36*	0.0 (-5.0 – 5.0)<br>*P=0.76*	0.0 (-5.0 – 5.0)<br>*P=0.77*	0.0 (0.0 – 5.0)<br>*P=0.56*	0.0 (-5.0 – 0.0)<br>*P=0.36*

Supplementary table I | EQ-5D-5L score at baseline, 24 hours, 2 weeks, 8 weeks, 6 months, 12 months follow-up. EQ-5D-5L: European Quality of Life 5-Dimension 5-Level, VAS: Visual Analogue Scale. PSA: Procedural sedation and analgesia, GA: General anesthesia. Medians with interquartile ranges, Hodges-Lehmann estimator for difference in median between groups with p-value for Mann-Whitney test. Sign-test for within group change from baseline.

Table J. UFS-QoL

UFS-QoL	Baseline		8 weeks	
	SSS	HRQL	SSS	HRQL
PSA	53.1 (40.6 – 68.8)<br>N=90	50.0 (37.9 – 69.0)<br>N=89	18.8 (9.4 – 31.3)<br>N=77	89.7 (77.2 – 98.3)<br>N=75
Change within group			-31.3 (-43.8 - -12.5)<br>*P=<0.0001*	25.9 (10.3 – 45.7)<br>*P=<0.0001*
GA	53.1 (35.9 – 62.5)<br>N=79	54.3 (39.7 – 78.0)<br>N=79	18.8 (9.4 – 34.4)<br>N=68	87.1 (74.6 – 93.5)<br>N=67
Change within group			-28.1 (-43.8 - -12.5)<br>*P=<0.0001*	20.7 (5.2 – 45.7)<br>*P=<0.0001*
Difference between groups			*0.0 (-6.3 – 3.1)*<br>*p=0.62*	*2.6 (-1.7 – 6.9)*<br>*p=0.26*

Supplementary Table J | Uterine Fibroid Symptom- Quality of Life questionnaire at baseline and 8 weeks follow-up. SSS: Symptom Severity Scale. HRQL: Health Related Quality of Life scale. PSA: Procedural sedation and analgesia, GA: General anesthesia. Medians with interquartile ranges, Hodges-Lehmann estimator for difference in median between groups with p-value for Mann-Whitney test. Sign-test for within group change from baseline

Appendix 3. Statistical Analysis Plan PROSECCO trial

The (cost) effectiveness of procedural sedation and analgesia versus general anesthesia for hysteroscopic myomectomy, a multicentre randomised controlled trial

Statistical Analysis Plan

**Author**	R.G. (Ruben) Duijnhoven
**Principal investigator, centre**	M.Y. (Marlies) Bongers, Maxima Medisch Centrum
**Coordinating investigator, centre**	H.S. (Helen) Kok, Alrijne Ziekenhuis
**Sponsor**	Máxima Medisch Centrum
**METC/CCMO approval no., approval date**	NL54779.015.15
**Funder**	ZonMW (843002603)

Table of contents

1. List of abbreviations ... iii
2. Index of tables ... iv
3. Index of figures ... iv
4. Changes compared to previous version ... v
5. Introduction ... 1
   5.1 Primary objective ... 1
   5.2 Secondary objectives ... 1
6. Patients ... 1
   6.1 Study population ... 1
   6.2 Inclusion criteria: ... 1
   6.3 Exclusion criteria ... 1
7. Study design ... 2
8. Randomisation and masking ... 2
9. Treatment of subjects ... 2
   9.1 Procedural sedation and analgesia ... 2
   9.2 General anesthesia ... 2
10. Endpoints ... 2
   10.1 Primary endpoint ... 2
   10.2 Secondary endpoint ... 2
   10.3 Other outcomes ... 3
11. Sample size and non-inferiority margin ... 3
12. General outline of analyses ... 3
   12.1 Trial profile ... 3
   12.2 Analysis populations ... 3
13. Planned analyses ... 3
   13.2 Covariates and subgroups ... 4
14. Missing data ... 5
15. Interim-analysis and data monitoring ... 5
16. Multiple testing ... 5
17. Presentation of study results – Tables ... 6
   17.1 Table 1. Baseline characteristics ... 6
   17.2 Table 2. Surgical procedure and anesthesia ... 7
   17.3 Table 3. Completeness of resection based on transvaginal ultrasound 6 weeks after hysteroscopic myomectomy ... 8
   17.4 Table 4. Surgeon’s judgement on completeness of procedure ... 9
   17.5 Table 5. Surgical complications ... 10
   17.6 Table 6. Anesthetic complications ... 11
   17.7 Table 7. Follow-up visit six weeks after surgery ... 12
   17.8 Table 8. Hospitalisation ... 13
   17.9 Table 9. Surgical reinterventions ... 13
   17.10 Table 10. Recovery Index Questionnaire ... 13
   17.11 Table 11. NRS ... 14
   17.12 Table 12. PBAC ... 14
   17.13 Table 13. UFS-QoL ... 14
   17.14 Table 14. EQ-5D-5L ... 15
18. Presentation of study results – Figures ... 15

18.1 Consort flowchart for study participants 16
19. References 17

1. List of abbreviations

AE	Adverse Event
AR	Adverse Reaction
CA	Competent Authority
CCMO	Central Committee on Research Involving Human Subjects / *Centrale Commissie Mensgebonden Onderzoek*
CRF	Case Record Form
DSMB	Data Safety Monitoring Board
EudraCT	European Clinical Trials Database
GCP	Good Clinical Practice
Hb	Haemoglobin
IMP	Investigational Medicinal Product
IMPD	Investigational Medicinal Product Dossier
METC	Research ethics committee / *Medisch Ethische Toetsingscommissie*
NAAP	Non-Anesthesiologist Administered Propofol
NRS	Numeric Rating Scale
PBAC	Pictorial Blood Assessment Chart
PSA	Procedural sedation and analgesia
RI-10	Recovery Index
SAE	Serious Adverse Event
SmPC	Summary of Product Characteristics
SUSAR	Suspected Unexpected Serious Adverse Reaction
TVU	Transvaginal Ultrasonography
UFS-QoL	Uterine Fibroid Symptoms – Quality of Life
Wbp	*Wet Bescherming Persoonsgevens*
WMO	*Wet Medisch-wetenschappelijk Onderzoek met Mensen*

2. Index of tables

Table 1 | Baseline characteristics ……………………………………………………………….... 6
Table 2 | Surgical procedure and anesthesia ……………………………………………………... 7
Table 3 | Completeness of resection based on transvaginal ultrasound 6 weeks after hysteroscopic myomectomy …………………………………………………………………………………….. 8
Table 4 | Surgeon's judgement on completeness of procedure ….…………………………………….. 9
Table 5 | Surgical complications ………………………………………………………………..... 10
Table 6 | Anesthetic complications ……………………………………………………………. 11
Table 7 | Follow-up visit six weeks after surgery …………………………………………………. 12
Table 8 | Hospitalisation ……………………………………………………………. 13
Table 9 | Surgical reinterventions ……………………………………………………………… 13
Table 10 | Recovery Index Questionnaire ………………………………………………………. 13
Table 11 | NRS …………………………………………………………………………………….. 14
Table 12 | PBAC ………………………………………………………………………………... 14
Table 13 | UFS-QoL ……………………………………………………………………………….. 14
Table 14 | EQ-5D-5L ……………………………………………………………………………. 15

3. Index of figures

Figure 1 | Consort flowchart for study participants 16

4. Changes compared to previous version

The current version is the second version.

Version	Date	Revision Details	Prepared	Checked	Approved
D	06 May 2020	Draft Issue	RD	JM / MB	n/a
1	29 Jan 2021	First accepted issue	RD / JM	JM / MB	RD/JM/MB/HK
2	22 Sep 2021	A longitudinal analysis was planned for UFS-Qol, EQ-5D-5L, RI and NRS scores, however this was not possible since data did not fit distribution assumptions. Planned analysis was changed: For those outcomes the Hodges-Lehmann estimator for difference in median between groups at the different time points with p-value for Mann-Whitney test was estimated. A sign test for within group change from baseline was used.	RD/JM	JM/MB	RD/JM/MB/HK

5. Introduction

Hysteroscopic myomectomies are performed in the majority of Dutch hospitals. The number of procedures for submucosal type 0 or I myomas between 1-3 cm performed in the operating room is estimated to be 3000 per year. Hysteroscopic myomectomy was traditionally performed in day treatment under general anesthesia.(1) A considerable cost reduction is expected when procedural sedation (PSA) is applied. The major contributing factors to the cost reduction are the shift from surgery in an operating theatre to an office-based setting, shorter hospital stay and outpatient care versus day care.(2,3) Higher patient satisfaction is expected, as hospital stay is shorter and side effects such as nausea are reduced. However, both safety and effectiveness – including the necessity for re-intervention due to incomplete resection – have not yet been fully evaluated.

5.1 Primary objective

To assess the non-inferiority in effectiveness of hysteroscopic resection of submucous myomas under procedural sedation with propofol (PSA) in an outpatient setting as compared to hysteroscopic myomectomy performed under general anesthesia in the operating room.

5.2 Secondary objectives

Secondary, completeness of resection as judged by the surgeon, pain, return to daily activities/recovery, duration of hospitalisation, (post)operative complications, the need for surgical re-interventions, menstrual blood loss (PBAC score), quality of life and uterine fibroids symptoms and related quality of life will be investigated.
In addition, economic analyses (cost-effectiveness) will be conducted, being outside the scope of this Statistical Analysis Plan.

6. Patients

6.1 Study population

Women 18 years or older, with a maximum of 3 symptomatic type 0 or I submucous myomas with a maximum diameter of 3.5 cm. They must be classified as American Society of Anesthesiologists class 1 or 2, and comprehension of the Dutch or English language to fully understand the study and to complete the questionnaires.

6.2 Inclusion criteria:

In order to be eligible to participate in this study, a subject must meet all of the following criteria:

- Minimum age of 18 years;
- Symptomatic type 0 or I submucous myomas
- Maximum number of submucous myomas of 3
- Maximum diameter of submucous myomas of 3.5 cm (as diagnosed by transvaginal ultrasonography)
- American Society of Anesthesiologist class 1 or 2

6.3 Exclusion criteria

The following exclusion criteria are used:

- American Society of Anesthesiologists class 3 or 4
- Presence of clotting disorders
- Severe anaemia (Hb under 5.0 mmol/L)
- Inability to understand Dutch or English

7. Study design

The study is designed as a multicentre open-label randomised controlled clinical trial to investigate non-inferiority of procedural sedation and analgesia with propofol as compared to general anesthesia.

8. Randomisation and masking

Randomisation is done using TEN/ALEA in a 1 : 1 ratio with variable block sizes of 2, 4, or 6, and is stratified by the surgical technique used (either morcellation or resection).

Healthcare providers and patients are not blinded.
The gynaecologist or ultrasonographer performing the transvaginal ultrasound 6 weeks after surgery will be blinded for the surgery outcome.

9. Treatment of subjects

Hysteroscopic resection is performed by an experienced surgeon by standard procedure in an office-based setting. Patients are observed after the procedure by qualified personnel and discharged as soon as all the discharge criteria are met, normally within 1 to 1.5 hours.

The way hysteroscopic resection is performed under general anesthesia does not differ from the way it is performed under procedural sedation.

9.1 Procedural sedation and analgesia

Patients randomised to procedural sedation and analgesia will receive propofol combined with alfentanil or remifentanil.
Sedation is given and monitored by a qualified sedation practitioner, according to guidelines from the Health Care Inspectorate (IGJ) and Dutch Institute for Healthcare Improvement (CBO) Non-Anesthesiologist Administered Propofol (NAAP).(4,5) The patient will be assessed by the sedation practitioner immediately prior to surgery on the basis of a preoperative questionnaire. Non-invasive blood pressure, electrocardiogram and oxygen saturation are measured before vascular access is obtained.

9.2 General anesthesia

General anesthesia can be inhalational or total intravenously, with the use of a laryngeal mask. Postoperatively, patients will be observed in the recovery room and discharged home from the clinic when all the discharge criteria are met.

10. Endpoints

10.1 Primary endpoint

Percentage of complete resections, as evaluated using transvaginal ultrasonography (TVU) (contrast sonography if TVU is inconclusive) six weeks postoperatively by an independent gynaecologist or ultrasonographer blinded for the treatment arm and surgery outcome.

A complete resection means that there are no signs of an intracavitary remaining of the fibroid (s) resected during hysteroscopic myomectomy. When resection is concluded to be incomplete based on the TVU, the images will be adjudicated by an independent review committee. When applicable the result of the TVU will be recorded as complete after adjudication.

10.2 Secondary endpoint

- Completeness of resection as judged by the surgeon during the procedure
- Pain (NRS score) when awake after the procedure and at discharge as recorded in the Case Record Form (CRF) and at 24 hours of follow-up as measured through a self-tailored questionnaire (supplementary file)
- Return to daily activities/recovery at 24 hours, 2 weeks and 8 weeks of follow-up as measured through the Recovery Index Questionnaire(6)

- Duration of hospitalisation in minutes as recorded in the CRF
- Peri- and postoperative complications until 6 weeks follow-up as recorded in the CRF
- The need for surgical re-interventions at 12 months follow-up as recorded in the CRF and measured through a self-tailored questionnaire (supplementary file)

10.3 Other outcomes

- Menstrual blood loss at baseline, 8 weeks and 12 months follow-up as measured through PBAC scores(7)
- Quality of life at baseline, 24 hours, 2 weeks, 8 weeks, 6 months, 12 months, as measured through the EQ-5D-5L questionnaire(8)
- Uterine fibroids symptoms and related quality of life at baseline and 8 weeks follow-up as measured through the UFS-QoL questionnaire(9,10)

11. Sample size and non-inferiority margin

The incidence of incomplete resections is estimated as 2.5% in both treatment groups based on expert opinion and previous literature.(11–13) An upper limit of the non-inferiority margin at 10% incomplete resections (i.e. a delta of 7.5%) is considered to represent noninferiority sufficiently. If alpha is chosen as 0.025 then a total sample size of 186 achieves 90% power. Allowing for a drop-out due to loss to follow-up of 10%, then 206 need to be recruited into the trial.

The sample size does not require adjustment for multiple testing, and interim analyses for efficacy are not conducted.

12. General outline of analyses

12.1 Trial profile

The flow of study participants will be displayed in a CONSORT diagram (Figure 1).

12.2 Analysis populations

12.2.1 Full Analysis population (ITT)

The Intention-To-Treat (ITT) populations will consist of all patients who have given consent and have been allocated one of the two treatments, irrespective of treatment received.

12.2.2 Per-protocol population

Secondary to the intention-to-treat population, the primary outcome will also be investigated using the per-protocol population.

The per protocol population is defined as any patient who underwent myomectomy with general anesthesia or procedural sedation as allocated using randomisation. Patients who did not have myomectomy or who did not receive the type of anesthesia allocated by randomisation will be removed from the per-protocol population.

13. Planned analyses

13.1.1 Baseline characteristics

Baseline characteristics and characteristics of surgical procedure and anesthesia will be presented as numbers and percentages in each group, or as averages (mean or median) with standard deviations or

interquartile ranges as appropriate. Relative risks will be given for surgical characteristics where applicable (Table 1,2).

13.1.2 Primary outcome

The primary outcome is complete myoma resection as determined using transvaginal ultrasonography six weeks after myomectomy.
The primary outcome will be evaluated by estimating the risk difference between both treatment arms. A Farrington-Manning test will be used to determine the p-value for non-inferiority.
Analyses will be conducted on both the intention-to-treat, as well as the per-protocol populations for the primary outcome (Table 3).

13.1.3 Secondary outcomes

Frequencies and relative risks for the surgeon's judgement on completeness of resection will be presented (Table 4).
The types, frequencies and relative risks for (post)operative complications will be given (Table 5-7).
Descriptive statistics on the duration of hospitalisation will be presented in table 8.
The types, frequencies and relative risks for surgical re-interventions after 12 months of follow-up will be given (Table 9)
Recovery Index scores at 24 hours, 2 weeks and 8 weeks and NRS scores for pain intensity after the procedure when awake, at discharge and at 24 hours will be reported as means with SD, or medians with IQR as appropriate, risk differences between both groups will be calculated with according 95% confidence interval (Table 10 and 11).

13.1.4 Other outcomes

PBAC scores at baseline, 8 weeks and 12 months will be analysed longitudinally using a generalized estimating equations model for repeated measures (Table 12).
UFS-QoL scores at baseline and 8 weeks and EQ-5D-5L mean total score and VAS scores at baseline, 24 hours, 2 weeks, 8 weeks, 6 months and 12 months are presented in table 13 and table 14. Differences in median between groups and changes from baseline within groups will be provided.

13.1.5 Statistical analysis

Relative risks will be estimated for categorical outcomes, with 95% confidence intervals, and χ^2 tests for significance. Fisher's exact test will be used for sparse events where assumptions of the χ^2 test are not met. Continuous data will be described as means with standard deviation if normally distributed, or medians with interquartile ranges when not normally distributed data; tests for significance are t-tests or Mann-Whitney U tests, respectively. The Hodges-Lehman estimator will be used to calculate the confidence interval for the difference in medians. A sign test for within group change from baseline will be used.
The PBAC scores will be analyzed longitudinally using generalized estimating equations.

13.2 Covariates and subgroups

13.2.1 Covariates

No analyses are planned for which estimates will be adjusted for effects of covariables other than the stratification factor (resection technique).
We will also calculate the relative risk, adjusted for the resection technique which was used.)

13.2.2 Subgroups

Analyses for the primary outcome will be performed by resection technique (resection or morcellation.

We will perform a subgroup analysis for fibroid size (< 2 cm versus ≥2 cm) and for parity (nulliparous versus multiparous women).

14. Missing data

The primary outcome is assessed six weeks after surgery, and much missing data for the primary outcome is not to be expected. However, drop-out has been allowed for in the sample size and a complete case analysis will be used if outcomes are missing. Imputation for missing data will not be used.

15. Interim-analysis and data monitoring

No interim analysis for efficacy will be conducted.

The DSMB charter of the study specifies interim safety review to be done after outcome data is available for every 50 participants. The DSMB may decide to alter the frequency of these safety reviews as needed.

16. Multiple testing

Given that interim analyses for efficacy are not conducted, correction for multiplicity of testing is considered not to be required.

Subgroup analyses will be done for parity and myoma size. These subgroup analyses will be exploratory. Adjustment of the p-value will not be done.

17. Presentation of study results – Tables

17.1 Table 1. Baseline characteristics

Baseline characteristics	Procedural sedation and analgesia N=NNN	General anesthesia N=NNN
Age, years (mean, sd)	xx.x (x.x)	xx.x (x.x)
BMI (mean, sd)	xx.x (x.x)	xx.x (x.x)
Gravidity (mean, sd)	xx.x (x.x)	xx.x (x.x)
Parity (mean, sd)	xx.x (x.x)	xx.x (x.x)
No. of vaginal deliveries	xx.x (x.x)	xx.x (x.x)
No. of caesarean sections	xx.x (x.x)	xx.x (x.x)
Previous uterine surgery	NN (%)	NN (%)
hysteroscopic myomectomy	NN (%)	NN (%)
laparoscopic myomectomy	NN (%)	NN (%)
embolization of myoma	NN (%)	NN (%)
sonography guided transcervical ablation of uterine fibroids	NN (%)	NN (%)
other	NN (%)	NN (%)
Use of hormonal medication at the time of hysteroscopic myomectomy	NN (%)	NN (%)
oral contraceptives	NN (%)	NN (%)
mirena IUD	NN (%)	NN (%)
implanon	NN (%)	NN (%)
oral progestogen (provera/orgametril)	NN (%)	NN (%)
nuvaRing	NN (%)	NN (%)
contraceptive injection (Depo Provera)	NN (%)	NN (%)
gnRH analogues	NN (%)	NN (%)
ulipristal acetate	NN (%)	NN (%)
Reason for hysteroscopic myomectomy		
abnormal uterine bleeding	NN (%)	NN (%)
abdominal complaints	NN (%)	NN (%)
subfertility	NN (%)	NN (%)
patients own wish after incidental finding of myoma	NN (%)	NN (%)
No. submucous myomas intended to be resected		
one	NN (%)	NN (%)
two	NN (%)	NN (%)
three	NN (%)	NN (%)
Myoma type		
type 0	NN (%)	NN (%)
type 1	NN (%)	NN (%)
Maximum size (diameter in mm; mean, sd)	xx.x (x.x)	xx.x (x.x)
Location		
anterior wall	NN (%)	NN (%)
posterior wall	NN (%)	NN (%)
fundus	NN (%)	NN (%)
left lateral wall	NN (%)	NN (%)
right lateral wall	NN (%)	NN (%)

cervical	NN (%)	NN (%)

Table 1 | Baseline characteristics. Sd: standard deviation, IUD: intrauterine device, GnRH: gonadotropin-releasing hormone. Percentages are column percentages based on the number of observations available (i.e. excluding missing observations).

17.2 Table 2. Surgical procedure and anesthesia

Surgical procedure and anesthesia	Procedural sedation N=NNN	General anesthesia N=NNN	Rel. risk (95% CI)	p-value
Anesthesia not according to randomisation:	NN (%)	NN (%)	x.xx (x.xx – x.xx)	0.xx
Reason:				
patient's own wish	NN (%)	NN (%)		
illness	NN (%)	NN (%)		
anesthesiologist's decision	NN (%)	NN (%)		
unknown	NN (%)	NN (%)		
Analgesics during procedural sedation				
alfentanil	NN (%)	n/a	n/a	-
remifentanil	NN (%)	n/a	n/a	-
Analgesics used prophylactically	NN (%)	NN (%)	x.xx (x.xx – x.xx)	0.xx
paracetamol	NN (%)	NN (%)		
NSAIDs	NN (%)	NN (%)		
opiate	NN (%)	NN (%)		
Procedure timed in first half of menstrual cycle	NN (%)	NN(%)	x.xx (x.xx – x.xx)	0.xx
Resection technique				
resectoscope	NN (%)	NN (%)	x.xx (x.xx – x.xx)	0.xx
morcellation	NN (%)	NN (%)	x.xx (x.xx – x.xx)	0.xx
Fluid loss, mL (median, IQR)	xxx (xxx – xxx)	xxx (xxx – xxx)	x.xx (x.xx – x.xx)†	0.xx
Time in surgery, minutes (mean, sd)	xx.x (xx.x)	xx.x (xx.x)	x.xx (xx.x – x.xx)*	0.xx
Presence of intracavitary polyp	NN (%)	NN (%)	x.xx (x.xx – x.xx)	0.xx
removed	NN (%)	NN (%)		

Table 2 | Surgical procedure and anesthesia. IQR: Interquartile Range, sd: standard deviation. Percentages are column percentages based on the number of observations available (i.e. excluding missing observations). * Mean difference and p-value for t-test † Difference in median with Hodges-Lehmann estimator and p-value for Mann-Whitney test.

17.3 Table 3. Completeness of resection based on transvaginal ultrasound 6 weeks after hysteroscopic myomectomy

Transvaginal ultrasonography six weeks after surgery								
	Intention to treat				Per protocol			
	Procedural sedation N=NNN	General anesthesia N=NNN	Rel. risk	P-value	Procedural sedation N=NNN	General anesthesia N=NNN	Rel. risk	P-value
Resection complete	NN (%)	NN (%)	x.xx (x.xx – x.xx)	0.xx	NN (%)	NN (%)	x.xx (x.xx – x.xx)	0.xx
When incomplete; max. size of intracavitary remnant (median, IQR)	xx (xx – xx)	xx (xx – xx)	x.xx (x.xx – x.xx)*	0.xx	xx (xx – xx)	xx (xx – xx)	x.xx (x.xx – x.xx)*	0.xx
<5 mm	NN (%)	NN (%)			NN (%)	NN (%)		
≥ 5 – 10 mm	NN (%)	NN (%)			NN (%)	NN (%)		
≥ 10 – 15 mm	NN (%)	NN (%)			NN (%)	NN (%)		
≥ 15 – 20 mm	NN (%)	NN (%)			NN (%)	NN (%)		
≥ 20 – 30 mm	NN (%)	NN (%)			NN (%)	NN (%)		
≥ 30 – 40 mm	NN (%)	NN (%)			NN (%)	NN (%)		
≥ 40 mm	NN (%)	NN (%)			NN (%)	NN (%)		
unknown	NN (%)	NN (%)			NN (%)	NN (%)		

Table 3| Completeness of resection based on transvaginal ultrasound 6 weeks after hysteroscopic myomectomy. IQR: Interquartile Range. Percentages are column percentages based on the number of observations available (i.e. excluding missing observations). * Difference in median (Hodges-Lehman), with p-value from Mann-Whitney test.

17.4 Table 4. Surgeon's judgement on completeness of procedure

Surgeon's judgement on completeness	Procedural sedation N=NNN	General anesthesia N=NNN	Rel. risk (95% CI)	p-value
Resection judged complete by surgeon	NN (%)	NN (%)	x.xx (x.xx – x.xx)	0.xx
Reason when incomplete:				
intramural extension of myoma	NN (%)	NN (%)		
amount of fluid loss	NN (%)	NN (%)		
poor visualization	NN (%)	NN (%)		
patient movement	NN (%)	NN (%)		
technical problems	NN (%)	NN (%)		
Surgeon's judgement on ease of procedure:				
easy no limiting factors	NN (%)	NN (%)	x.xx (x.xx – x.xx)	0.xx
view/access limited, performance not influenced	NN (%)	NN (%)		
view/access limited, influencing performance	NN (%)	NN (%)		
not possible to perform procedure	NN (%)	NN (%)		
When incomplete, need for 2nd session	NN	NN (%)		
complete	NN (%)	NN (%)		
When incomplete, need for 3rd session	NN (%)	NN (%)		
complete	NN (%)	NN (%)		

Table 4 | Surgeon's judgement on completeness of procedure. Percentages are column percentages based on the number of observations available (i.e. excluding missing observations).

17.5 Table 5. Surgical complications

Surgical complications	Procedural sedation N=NNN	General anesthesia N=NNN	Rel. risk (95% CI)	p-value
Surgical complications	NN (%)	NN (%)	x.xx (x.xx – x.xx)	0.xx
perforation of uterine wall	NN (%)	NN (%)		
bowel injury	NN (%)	NN (%)		
thermal injury	NN (%)	NN (%)		
extravasation resulting in electrolyte disturbances	NN (%)	NN (%)		
haemorrhage	NN (%)	NN (%)		
abdominal haemorrhage	NN (%)	NN (%)		
cervical laceration	NN (%)	NN (%)		
other	NN (%)	NN (%)		
Treatment of complication	NN (%)	NN (%)	x.xx (x.xx – x.xx)	0.xx
If treated, specification:				
medication given	NN (%)	NN (%)		
antibiotics	NN (%)	NN (%)		
pain medication	NN (%)	NN (%)		
diuretics	NN (%)	NN (%)		
extension of admission (overnight stay)	NN (%)	NN (%)		
need for immediate surgery	NN (%)	NN (%)		
blood transfusion	NN (%)	NN (%)		
intensive care admission	NN (%)	NN (%)		
other	NN (%)	NN (%)		

Table 5 | Surgical complications. Percentages are column percentages based on the number of observations available (i.e. excluding missing observations).

17.6 Table 6. Anesthetic complications

Anesthetic complications	Procedural sedation N=NNN	General anesthesia N=NNN	Rel. risk (95% CI)	P-value
Anesthetic complication needing intervention	NN (%)	NN (%)	x.xx (x.xx – x.xx)	0.xx
if complication, specification:				
Desaturation	NN (%)	NN (%)		
airway obstruction	NN (%)	NN (%)		
Dysrhythmias	NN (%)	NN (%)		
hypotension	NN (%)	NN (%)		
ischaemic ECG alterations (ST deviation)	NN (%)	NN (%)		
difficult intubation	NN (%)	NN (%)		
vomiting / aspiration	NN (%)	NN (%)		
perioperative nausea / vomiting	NN (%)	NN (%)		
other	NN (%)	NN (%)		
Treatment of complication:				
supplemental oxygen	NN (%)	NN (%)		
airway manoevre	NN (%)	NN (%)		
naso-/oropharyngeal airway	NN (%)	NN (%)		
bag valve mask assisted ventilation	NN (%)	NN (%)		
laryngeal mask	NN (%)	NN (%)		
Intubation	NN (%)	NN (%)		
rapid i.v. fluids	NN (%)	NN (%)		
medication given:	NN (%)	NN (%)		
diuretics	n	n		
fenylefrine	n	n		
ephedrine	n	n		
atropine	n	n		
naloxone	n	n		

Table 6 | Anesthetic complications. Percentages are column percentages based on the number of observations available (i.e. excluding missing observations).

17.7 Table 7. Follow-up visit six weeks after surgery

Follow-up visit six weeks after surgery	Procedural sedation N=NNN	General anesthesia N=NNN	Rel. risk N=NNN	P-value
Complications between discharge and follow-up visit	NN (%)	NN (%)	x.xx (x.xx – x.xx)	0.xx
If complication, specification				
infection	NN (%)	NN (%)		
hemorrhage	NN (%)	NN (%)		
pain	NN (%)	NN (%)		
other	NN (%)	NN (%)		
Treatment of complication	NN (%)	NN (%)	x.xx (x.xx – x.xx)	0.xx
medication	NN (%)	NN (%)		
need for new appointment	NN (%)	NN (%)		
need for re-operation	NN (%)	NN (%)		
other	NN (%)	NN (%)		
Pathological diagnosis leiomyoma	NN (%)	NN (%)	x.xx (x.xx – x.xx)	0.xx
diagnosis if not leiomyoma: polyp	NN (%)	NN (%)		
other	NN (%)	NN (%)		
Postoperative use of hormonal medication	NN (%)	NN (%)	x.xx (x.xx – x.xx)	0.xx
Continuation of preoperative medication	NN (%)	NN (%)	x.xx (x.xx – x.xx)	0.xx
Started postoperatively	NN (%)	NN (%)	x.xx (x.xx – x.xx)	0.xx
oral contraceptives	NN (%)	NN (%)		
mirena IUD	NN (%)	NN (%)		
implanon	NN (%)	NN (%)		
oral progestogen	NN (%)	NN (%)		
nuvaRing	NN (%)	NN (%)		
contraceptive injection	NN (%)	NN (%)		
GnRH analogues	NN (%)	NN (%)		
ulipristal acetate	NN (%)	NN (%)		

Table 7 | Follow-up visit six weeks after surgery. Percentages are column percentages based on the number of observations available (i.e. excluding missing observations).

17.8 Table 8. Hospitalisation

Hospitalisation	Procedural sedation N=NNN	General anesthesia N=NNN	Rel. risk (95% CI)	p-value
Duration of admission to the hospital, minutes(mean, sd)	xx.x (xx.x)	xx.x (xx.x)	x.xx (xx.x – x.xx)*	0.xx
Time until Aldrete score ≥ 9 after procedure, min (mean, sd)	xx.x (xx.x)	xx.x (xx.x)	x.xx (xx.x – x.xx)*	
> 2 hours between discharge and Aldrete score ≥ 9	NN (%)	NN (%)	x.xx (x.xx – x.xx)	0.xx
Reason:				
logistics	NN (%)	NN (%)		
complications	NN (%)	NN (%)		
not fit enough	NN (%)	NN (%)		
other	NN (%)	NN (%)		
unknown	NN (%)	NN (%)		

Table 8 | Hospitalisation IQR: Interquartile Range. Percentages are column percentages based on the number of observations available (i.e. excluding missing observations). * Mean difference and p-value for t-test

17.9 Table 9. Surgical reinterventions

Recurrence 12 months after surgery	Procedural sedation N=NNN	General anesthesia N=NNN	Rel. risk	P-value
Recurrence of symptoms	NN (%)	NN (%)	x.xx (x.xx – x.xx)	0.xx
Re-intervention	NN (%)	NN (%)	x.xx (x.xx – x.xx)	0.xx
Medication	NN (%)	NN (%)		
Hysteroscopic myomectomy	NN (%)	NN (%)		
Endometrial ablation	NN (%)	NN (%)		
Hysterectomy	NN (%)	NN (%)		

Table 9 | Surgical reinterventions 12 months after surgery. Percentages are column percentages based on the number of observations available.

17.10 Table 10. Recovery Index Questionnaire

RI10 score	24 hours	2 weeks	8 weeks
PSA	Med (IQR), N	Med (IQR), N	Med (IQR), N
GA	Med (IQR), N	Med (IQR), N	Med (IQR), N
Difference between groups	… (95% CI) *P=XX*	… (95% CI) *P=XX*	… (95% CI) *P=XX*

Table 10 | RI10 score at 24 hours, 2 weeks and 8 weeks follow-up. RI10: Recovery Index Questionnaire. PSA: Procedural sedation and analgesia, GA: general anesthesia. Medians with interquartile ranges. Hodges-Lehmann estimator for difference in median between groups with p-value for Mann-Whitney test.

17.11 Table 11. NRS

NRS score	Recovery room	Discharge	24 hours
PSA	Med (IQR), N	Med (IQR), N	Med (IQR), N
GA	Med (IQR), N	Med (IQR), N	Med (IQR), N
Difference between groups	… (95% CI) *P=XX*	… (95% CI) *P=XX*	… (95% CI) *P=XX*

Table 11 | NRS score at return in recovery room, discharge and 24 hours follow-up. NRS: Numeric Rating Scale. PSA: Procedural sedation and analgesia, GA: General anesthesia. Medians with interquartile ranges. Hodges-Lehmann estimator for difference in median between groups with p-value for Mann-Whitney test.

17.12 Table 12. PBAC

PBAC score	Baseline	8 weeks	12 months	Overall*
General anesthesia	Mean (SD), n	Mean (SD), n	Mean (SD), n	
Change within group		… (95% CI) *P=XX*	… (95% CI) *P=XX*	
Procedural sedation	Mean (SD), n	Mean (SD), n	Mean (SD), n	
Change within group		… (95% CI) *P=XX*	… (95% CI) *P=XX*	
Difference between groups		… (95% CI) *P=XX*	… (95% CI) *P=XX*	… (95% CI) *P=XX*

Table 12 | PBAC score at baseline, 8 weeks, 12 months follow-up. PBAC: Pictorial Blood Assessment Chart. *Overall estimate is mean difference over all time points using a repeated measures model including variables adjusting for group, baseline score and time.

17.13 Table 13. UFS-QoL

UFS-QoL	Baseline		8 weeks	
	SSS	HRQL	SSS	HRQL
PSA	Med (IQR), n	Med (IQR), n	Med (IQR), n	Med (IQR), n
Change within group			… (95% CI) *P=XX*	… (95% CI) *P=XX*
General anesthesia	Med (IQR), n	Med (IQR), n	Med (IQR), n	Med (IQR), n
Change within group			… (95% CI) *P=XX*	… (95% CI) *P=XX*
Difference between groups			… (95% CI) *P=XX*	… (95% CI) *P=XX*

Table 13 | Uterine Fibroid Symptom- Quality of Life questionnaire at baseline and 8 weeks follow-up. SSS: Symptom Severity Scale. HRQL: Health Related Quality of Life scale. PSA: Procedural sedation and analgesia, GA: General anesthesia. Medians with interquartile ranges, Hodges-Lehmann estimator for difference in median between groups with p-value for Mann-Whitney test. Sign-test for within group change from baseline.

17.14 Table 14. EQ-5D-5L

EQ-5D-5L score	Baseline	24 hours	2 weeks	8 weeks	6 months	12 months
General anesthesia	Med (IQR), n	Med (IQR), n	Med (IQR), n	Med (IQR), n	Med (IQR), n	Med (IQR), n
Change within group		…(95% CI) *P=XX*	…(95% CI) *P=XX*	…(95%CI) *P=XX*	… (95% CI) *P=XX*	… (95% CI) *P=XX*
Procedural sedation	Mean (SD), n	Med (IQR), n	Med (IQR), n	Med (IQR), n	Med (IQR), n	Med (IQR), n
Change within group		…(95% CI) *P=XX*	…(95% CI) *P=XX*	…(95%CI) *P=XX*	… (95% CI) *P=XX*	… (95% CI) *P=XX*
Difference between groups		…(95% CI) *P=XX*	…(95% CI) *P=XX*	…(95%CI) *P=XX*	… (95% CI) *P=XX*	… (95% CI) *P=XX*

EQ-5D-5L VAS score	Baseline	24 hours	2 weeks	8 weeks	6 months	12 months
General anesthesia	Med (IQR), n	Med (IQR), n	Med (IQR), n	Med (IQR), n	Med (IQR), n	Med (IQR), n
Change within group		…(95% CI) *P=XX*	…(95% CI) *P=XX*	…(95%CI) *P=XX*	… (95% CI) *P=XX*	… (95% CI) *P=XX*
Procedural sedation	Med (IQR), n	Med (IQR), n	Med (IQR), n	Med (IQR), n	Med (IQR), n	Med (IQR), n
Change within group		…(95% CI) *P=XX*	…(95% CI) *P=XX*	…(95%CI) *P=XX*	… (95% CI) *P=XX*	… (95% CI) *P=XX*
Difference between groups		…(95% CI) *P=XX*	…(95% CI) *P=XX*	…(95%CI) *P=XX*	… (95% CI) *P=XX*	… (95% CI) *P=XX*

Table 14 | EQ-5D-5L score at baseline, 24 hours, 2 weeks, 8 weeks, 6 months, 12 months follow-up. EQ-5D-5L: European Quality of Life 5-Dimension 5-Level, VAS: Visual Analogue Scale, Med: Median, IQR: interquartile range. Medians with interquartile ranges, Hodges-Lehmann estimator for difference in median between groups with p-value for Mann-Whitney test. Sign-test for within group change from baseline.

18. Presentation of study results – Figures

18.1 Consort flowchart for study participants

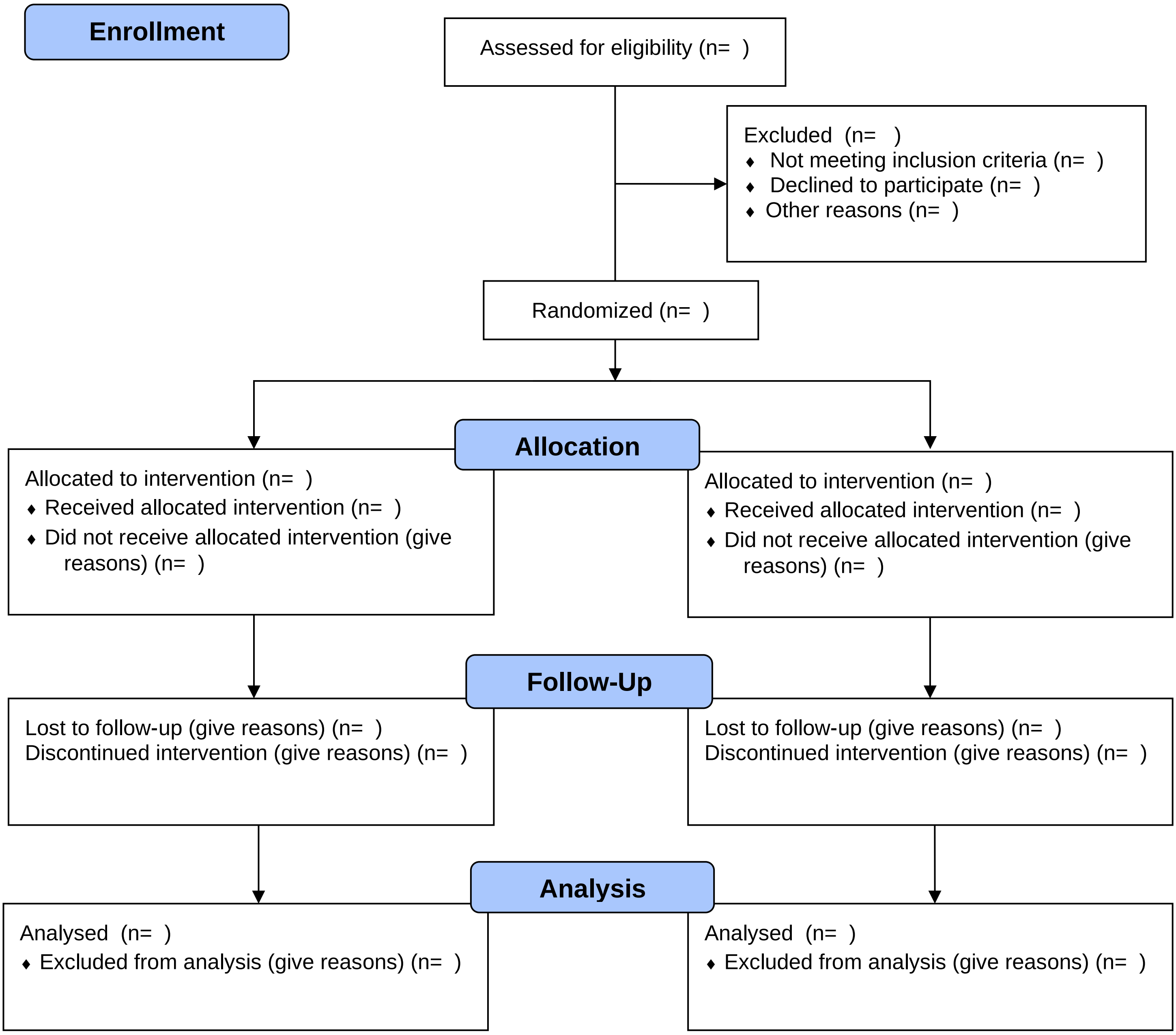

19. References

1. Di Spiezio Sardo A, Mazzon I, Bramante S, Bettocchi S, Bifulco G, Guida M, et al. Hysteroscopic myomectomy: a comprehensive review of surgical techniques. Hum Reprod Update. 2008;14(2):101–19.
2. Marsh F, Kremer C, Duffy S. Delivering an effective outpatient service in gynaecology. A randomised controlled trial analysing the cost of outpatient versus daycase hysteroscopy. BJOG An Int J Obstet Gynaecol. 2004 Mar;111(3):243–8.
3. Kremer C, Duffy S, Moroney M. Patient satisfaction with outpatient hysteroscopy versus day case hysteroscopy: randomised controlled trial. BMJ. 2000 Jan 29;320(7230):279–82.
4. Kwaliteitsinstituut voor de Gezondheidszorg CBO. Richtlijn sedatie en/of analgesie (PSA) op locaties buiten de operatiekamer. 2009.
5. Inspectie voor de Gezondheidszorg. Toetsingskader sedatie en of analgesie buiten de operatiekamer 2012. Available from: http://www.google.nl/url?sa=t&rct=j&q=&esrc=s&source=web&cd=1&cad=rja&ved=0CDIQFjAA&url=http://www.igz.nl/Images/toetsingskader sedatie _tcm294-328212.pdf&ei=YcqpUfg3qMDRBY2TgJgO&usg=AFQjCNE4moBXDfYJ68GUhT8lniFytrZYYg&sig2=WSx-w6oXGVMSCYZtQotztw
6. Kluivers KB, Hendriks JCM, Mol BWJ, Bongers MY, Vierhout ME, Brölmann HAM, et al. Clinimetric properties of 3 instruments measuring postoperative recovery in a gynecologic surgical population. Surgery. 2008;144(1):12–21.
7. Higham JM, O'Brien PMS, Shaw RW. Assessment of menstrual blood loss using a pictorial chart. BJOG An Int J Obstet Gynaecol. 1990 Aug;97(8):734–9.
8. Herdman M, Gudex C, Lloyd A, Janssen M, Kind P, Parkin D, et al. Development and preliminary testing of the new five-level version of EQ-5D (EQ-5D-5L). Qual Life Res. 2011;20(10):1727–36.
9. Spies JB, Coyne K, Guaou Guaou N, Boyle D, Skyrnarz-Murphy K, Gonzalves SM. The UFS-QOL, a new disease-specific symptom and health-related quality of life questionnaire for leiomyomata. Obstet Gynecol. 2002;99(2):290–300.
10. Keizer AL, van Kesteren PJM, Terwee C, de Lange ME, Hehenkamp WJK, Kok HS. Uterine Fibroid Symptom and Quality of Life questionnaire (UFS-QOL NL) in the Dutch population: a validation study. BMJ Open. 2021 Nov 23;11(11):e052664.
11. Litta P, Leggieri C, Conte L, Dalla Toffola A, Multinu F, Angioni S. Monopolar versus bipolar device: Safety, feasibility, limitsand perioperative complications in performing hysteroscopic myomectomy. Clin Exp Obstet Gynecol. 2014;41(3):335–8.
12. Lasmar RB, Xinmei Z, Indman PD, Celeste RK, Di Spiezio Sardo A. Feasibility of a new system of classification of submucous myomas: a multicenter study. Fertil Steril. 2011 May;95(6):2073–7.
13. Muzii L, Boni T, Bellati F, Marana R, Ruggiero A, Zullo M a, et al. GnRH analogue treatment before hysteroscopic resection of submucous myomas: a prospective, randomized, multicenter study. Fertil Steril. 2010 Sep;94(4):1496–9.
